# Mesodermal FGF and BMP govern the sequential stages of zebrafish thyroid specification

**DOI:** 10.1242/dev.201023

**Published:** 2023-05-16

**Authors:** Benoit Haerlingen, Robert Opitz, Isabelle Vandernoot, Angelo Molinaro, Meghna Parakkal Shankar, Pierre Gillotay, Achim Trubiroha, Sabine Costagliola

**Affiliations:** ^1^ Institute of Interdisciplinary Research in Molecular Human Biology (IRIBHM), Université Libre de Bruxelles, 1070 Brussels, Belgium; ^2^ Institute of Experimental Pediatric Endocrinology, Charité Universitätsmedizin, 13353 Berlin, Germany; ^3^ Centre of Human Genetics, Erasmus Hospital, Université Libre de Bruxelles, 1070 Brussels, Belgium; ^4^ Department of Experimental and Clinical Medicine, Endocrinology Unit, University of Pisa, 56126 Pisa, Italy; ^5^ Department Chemicals and Product Safety, German Federal Institute for Risk Assessment (BfR), 10589 Berlin, Germany

**Keywords:** Zebrafish, Endoderm, Thyroid, Specification, FGF, BMP

## Abstract

Thyroid tissue, the site of *de novo* thyroid hormone biosynthesis, is derived from ventral pharyngeal endoderm and defects in morphogenesis are a predominant cause of congenital thyroid diseases. The first molecularly recognizable step of thyroid development is the specification of thyroid precursors in anterior foregut endoderm. Recent studies have identified crucial roles of FGF and BMP signaling in thyroid specification, but the interplay between signaling cues and thyroid transcription factors remained elusive. By analyzing Pax2a and Nkx2.4b expression dynamics in relation to endodermal FGF and BMP signaling activities in zebrafish embryos, we identified a Pax2a-expressing thyroid progenitor population that shows enhanced FGF signaling but lacks Nkx2.4b expression and BMP signaling. Concurrent with upregulated BMP signaling, a subpopulation of these progenitors subsequently differentiates into lineage-committed thyroid precursors co-expressing Pax2a and Nkx2.4b. Timed manipulation of FGF/BMP activities suggests a model in which FGF signaling primarily regulates Pax2a expression, whereas BMP signaling regulates both Pax2a and Nkx2.4b expression. Our observation of similar expression dynamics of Pax8 and Nkx2-1 in mouse embryos suggests that this refined model of thyroid cell specification is evolutionarily conserved in mammals.

## Teaser

**Summary:** Specification of the early thyroid anlage involves a Pax2a-expressing thyroid progenitor cell population that is subject to sequential induction of Pax2a and Nkx2.4b, mediated by differential FGF- and BMP-dependent patterning processes.

## INTRODUCTION

The essential role of the thyroid gland is to produce thyroid hormones (THs), which regulate key processes in embryonic development and adult homeostasis (Bernal, 2007; Buchholz et al., 2006; Mullur et al., 2014). The functional subunits of thyroid tissue are the TH-producing follicles consisting of a single-layer epithelium of thyroid follicular cells (TFCs). Deficiencies in TH synthesis at birth cause congenital hypothyroidism (CH) with about 85% of CH cases being a result of thyroid dysgenesis (TD), but the genetic origin of CH due to TD is known in <5% of cases (Wassner, 2018). It is remarkable that there is an increased prevalence of cardiovascular malformations in people with TD (Olivieri et al., 2002), implying a possible mechanistic relationship between developmental abnormalities of the thyroid and the cardiovascular system.

Thyroid morphogenesis has been studied intensively in murine and zebrafish models (reviewed by Nilsson and Fagman, 2017; Opitz et al., 2013; Porazzi et al., 2009). The first molecularly recognizable event of TFC differentiation is the specification of thyroid precursors in the anterior foregut endoderm. These thyroid lineage-committed precursors are characterized by co-expression of key thyroid transcription factors Nkx2-1 and Pax8 in mammals, and Nkx2.4b and Pax2a in zebrafish (Kimura, 1996; Mansouri et al., 1998; Rohr and Concha, 2000; Wendl et al., 2002). Shortly after specification, the thyroid anlage undergoes a complex series of morphogenetic events, such as budding, relocalization, folliculogenesis and functional maturation, including the induction of functional differentiation markers such as thyroglobulin, thyroid peroxidase or the sodium-iodide symporter (Alt et al., 2006; Fagman and Nilsson, 2011; Fagman et al., 2006; Opitz et al., 2011, 2012).

Compared with other endoderm-derived organs (Bastidas-Ponce et al., 2017; Ober and Lemaigre, 2018), the molecular mechanisms underlying thyroid specification are still poorly understood, in particular how extrinsic signaling and intrinsic factors orchestrate the earliest steps of thyroid cell differentiation. Recent studies have highlighted the important roles of fibroblast growth factor (FGF) and bone morphogenetic protein (BMP) signaling. Murine mutant models with defective FGF signaling display severe thyroid hypoplasia or absence of thyroid tissue (Celli et al., 1998; Ohuchi et al., 2000; Revest et al., 2001) and the importance of this pathway for thyroid specification has since been confirmed in various *in vitro* and *in vivo* models (Haerlingen et al., 2019; Kurmann et al., 2015; Longmire et al., 2012; Serls et al., 2005; Wendl et al., 2007). In stem cell-based models, FGF2 stimulation of anterior foregut endoderm cell cultures promotes differentiation of thyroid precursors (Kurmann et al., 2015). Conversely, pharmacological inhibition of FGF signaling blocks thyroid specification in mouse foregut explants and *Xenopus* and zebrafish embryos. BMP activity is another crucial signaling pathway. For example, BMP4 is a potent stimulator of thyroid precursor differentiation in *in vitro* stem cell models and blocking BMP signaling during somitogenesis causes a lack of thyroid specification in *Xenopus* and zebrafish embryos (Haerlingen et al., 2019; Kurmann et al., 2015).

Observations made in different species consistently point to precardiac and cardiac mesoderm as a major source of FGF and BMP ligands acting on the foregut endoderm to initiate thyroid cell differentiation (Serls et al., 2005; Vandernoot et al., 2020; Wendl et al., 2007). Fate-mapping analyses of early zebrafish endoderm revealed that TFCs are derived from a portion of endoderm positioned at the midbrain–hindbrain boundary level just medial to the precardiac anterior lateral plate mesoderm (LPM) (Wendl et al., 2007). In all species analyzed, the thyroid anlage is specified in the region of the foregut endoderm that is immediately adjacent to the cardiac mesoderm forming the outflow tract of the developing heart (Alt et al., 2006; Haerlingen et al., 2019; Norris, 1920; Wendl et al., 2007). Zebrafish studies also showed that loss of cardiac mesoderm, impaired myocardial differentiation or perturbed positioning of cardiac mesoderm relative to the prospective thyroid field in the foregut endoderm results in aberrant thyroid development (Haerlingen et al., 2019; Vandernoot et al., 2020).

Here, we used zebrafish embryos to map expression dynamics of Pax2a and Nkx2.4b spatiotemporally as well as FGF and BMP signaling activities in the prospective thyroid field of the foregut endoderm. We identified an endodermal thyroid progenitor cell population expressing Pax2a and report for the first time that the early thyroid anlage forms by sequential induction of Pax2a and Nkx2.4b. Moreover, we show that this sequence of transcription factor induction mirrors the onset of FGF and BMP signaling activity within the anterior foregut endoderm. Timed manipulation of FGF and BMP signaling revealed that FGF signaling primarily regulates Pax2a expression, whereas BMP signaling has complex functions in regulation of both Pax2a and Nkx2.4b expression. By integrating our data with results from previous studies, we propose a refined model of thyroid cell specification that explains the specific positioning of the thyroid anlage as a result of FGF- and BMP-dependent patterning processes. Corresponding analyses of early mouse foregut development show that the sequential induction of Pax8 and Nkx2-1 expression is an evolutionarily conserved mechanism during thyroid anlage specification.

## RESULTS

### Sequential induction of the thyroid transcription factors Pax2a and Nkx2.4b

The differentiation of ventral foregut endodermal cells into thyroid precursors is the earliest recognizable step of thyroid morphogenesis and occurs around embryonic day (E) 8.5 in mice (co-expression of *Nkx2-1* and *Pax8*) and around 23/24 hours post-fertilization (hpf) in zebrafish (co-expression of *nkx2.4b* and *pax2a*) (Fagman et al., 2006; Wendl et al., 2002). Although it is commonly accepted that co-expression of these transcription factors is a hallmark of lineage commitment, the induction dynamics and possible molecular mechanisms of thyroid cell specification remained largely unexplored.

**Fig. 1.**
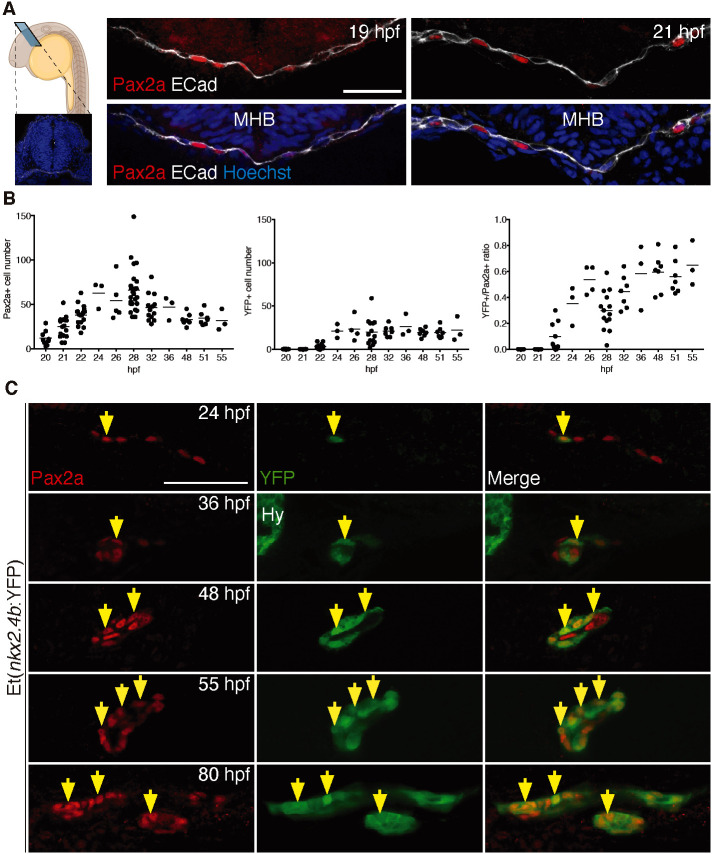
**Sequential induction of thyroid transcription factors in foregut endoderm.** (A) Panel on the left shows a schematic of a zebrafish embryo depicting the sectioning plane and a cross-section highlighting the anatomical organization of the region analyzed; E-cadherin (Cadherin 1; white) labels epithelia and DAPI (blue) cell nuclei. Panels on the right show Pax2a expression in anterior foregut endoderm (labeled with E-cadherin) before thyroid anlage formation. Confocal images of cross-sections at the level of the MHB are shown. (B) Total number of foregut endodermal cells expressing Pax2a (left) and YFP (middle) in Et(*nkx2.4b*:YFP) embryos and proportion of Pax2a^+^/YFP^+^ cells within the Pax2a^+^ cell population (right). Values determined in individual embryos (dots) and mean values (bars) are shown. (C) Pax2a and YFP expression in thyroid region of Et(*nkx2.4b*:YFP) embryos. Confocal images of sagittal (24-55 hpf) and coronal sections (80 hpf) are shown with arrows marking selected cells co-expressing Pax2a and YFP. Anterior is to the left. Hy, YFP expression in hypothalamus. Scale bars: 25 µm.

To characterize Pax2a induction in the presumed zebrafish thyroid field, we performed immunofluorescence (IF) staining on a series of wild-type (WT) embryos sampled every 2 h from 15/16 hpf. Using a highly specific anti-Pax2a antibody (Trubiroha et al., 2018), endodermal Pax2a expression became detectable by 18/19 hpf in a region ventral to the midbrain–hindbrain boundary (MHB) (Fig. 1A). By 20 hpf, already up to 30 cells with strong Pax2a expression were detectable (Fig. 1B) and their number increased rapidly to reach peak levels at 28 hpf. The rostral edge of the Pax2a expression domain was located ventral to the MHB and the domain extended caudally up to 100 µm along the anteroposterior (AP) axis in 22 hpf embryos. Notably, the foregut of 28 hpf embryos contained up to 100 Pax2a^+^ cells, whereas the thyroid primordium of 55 hpf embryos would eventually comprise only 30-40 differentiated thyroid cells (Trubiroha et al., 2018). Indeed, we observed a progressive decline in the number of Pax2a^+^ endodermal cells between 28 and 55 hpf (see Fig. 1B).

To overcome the lack of a specific anti-Nkx2.4b antibody, we monitored Nkx2.4b expression using the enhancer trap line Et(*nkx2.4b*:YFP) (Ellingsen et al., 2005). In this line, YFP expression is regulated by the *nkx2.4b* promoter and the spatiotemporal YFP reporter expression correlates neatly with *nkx2.4b* mRNA expression during early zebrafish development (see Fig. S1 for *nkx2.4b* mRNA expression profile). In the foregut endoderm region, we detected neither YFP expression (Fig. S1B) by IF nor endogenous *nkx2.4b* mRNA (Fig. S1A) by whole-mount *in situ* hybridization (WISH) prior to 22 hpf. Faint YFP staining of endodermal cells was first detectable in a few 22 hpf embryos and it was only from 23 hpf onwards that YFP^+^ cells were reliably detected in the foregut endoderm (Fig. 1B,C). Corresponding observations about the onset of *nkx2.4b* mRNA expression were made by *nkx2.4b* WISH (Fig. S1A).

Co-staining of Pax2a and YFP in Et(*nkx2.4b*:YFP) embryos showed that YFP expression is exclusively detectable in Pax2a^+^ cells (Fig. 1C), at all analyzed stages. Conversely, only a fraction of Pax2a^+^ endodermal cells showed co-expression of the *nkx2.4b*:YFP reporter during thyroid anlage formation. The ratio of YFP^+^/Pax2a^+^ cells fluctuated over time, mainly owing to variation in the number of Pax2a^+^ cells.

Collectively, our analyses showed that the onset of endodermal *nkx2.4b* expression is delayed by several hours relative to Pax2a, demonstrating for the first time that zebrafish thyroid specification is a multi-step process. The initial step in this process is the induction of an as-yet-unrecognized population of Pax2a^+^ foregut progenitor cells from which only a subpopulation will be specified into committed Pax2a^+^/Nkx2.4b^+^ thyroid precursors as a second step.

### Sequential FGF and BMP signaling activities during thyroid specification

The sequential appearance of Pax2a^+^ thyroid progenitors and committed Pax2a^+^/Nkx2.4b^+^ thyroid precursors raises the question of possible extrinsic signals regulating these processes. Recently, we showed that FGF and BMP signaling are crucial pathways regulating early zebrafish thyroid development (Haerlingen et al., 2019). To monitor FGF signaling activities, we used the Tg(*dusp6*:d2EGFP) FGF signaling biosensor line expressing a destabilized EGFP version under the control of the FGF-responsive *dusp6* (previously known as *mkp3*) promoter (Molina et al., 2007; Tsang et al., 2004). For analysis of BMP signaling activities, we used the Tg(BRE:dmKO2) BMP signaling biosensor line (Collery and Link, 2011), which carries a synthetic BMP-responsive element (BRE containing multiple binding sites for phosphorylated Smad proteins) that drives expression of destabilized monomeric Kusabira-Orange 2 (dmKO2). The use of destabilized fluorescent reporters in both lines provided for improved temporal signal resolution. Both biosensor lines have been previously used to monitor endogenous BMP and FGF signaling activities and their responsiveness to experimental inhibition or overactivation of signaling activities is well documented (Lombardo et al., 2019; Roussigné et al., 2018; Sheehan-Rooney et al., 2013).

**Fig. 2.**
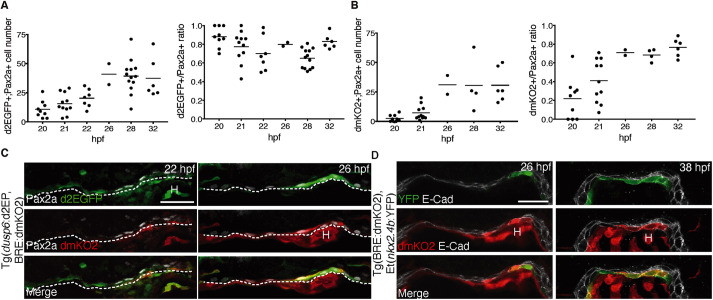
**Sequential appearance of FGF and BMP signaling in Pax2-expressing endoderm.** (A) FGF signaling reporter expression. Left: Total number of Pax2a^+^/d2GFP^+^ cells in foregut endoderm of Tg(*dusp6*:d2EGFP) embryos. Right: Relative abundance of Pax2a^+^/d2GFP^+^ cells in the Pax2a^+^ cell population. Values determined in individual embryos (dots) and mean values (bars) are shown. (B) BMP signaling reporter expression. Left: Total number of Pax2a^+^/dmKO2^+^ cells in foregut endoderm of Tg(BRE:dmKO2) embryos. Right: Relative abundance of Pax2a^+^/dmKO2^+^ cells in the Pax2a^+^ cell population. (C) Pax2a, d2GFP and dmKO2 expression in thyroid region of Tg(*dusp6*:d2EGFP;BRE:dmKO2) double-transgenic embryos. Confocal images of transverse sections. Dashed line marks border between endoderm and ventral foregut mesenchyme. (D) IF of E-cadherin (E-Cad), dmKO2 and YFP expression in thyroid region of Tg(BRE:dmKO2);Et(*nkx2.4b:*YFP) double-transgenic embryos. Confocal images of transverse sections. Note that YFP expression is restricted to endodermal cells expressing the BMP signaling reporter dmKO2. H, heart. Scale bars: 25 µm.

When monitoring FGF signaling in the foregut endoderm, the first detectable d2EGFP signals could be observed in 18/19 hpf embryos (Fig. S2). FGF signaling reporter expression in the endoderm was largely confined to Pax2a^+^ cells and most Pax2a^+^ cells showed d2EGFP expression (Fig. S2). Quantitative analysis of embryos sampled between 20 and 32 hpf showed that a relatively stable proportion of about 80% of Pax2a^+^ thyroid progenitors displayed a robust d2EGFP signal (Fig. 2), linking for the first time FGF activity in the foregut endoderm to Pax2a expression. Notably, Pax2a-negative cells with detectable d2EGFP signal were rarely observed in the foregut endoderm.

In contrast, when monitoring BMP signaling in the thyroid region, we found that dmKO2 expression was largely absent in the endoderm prior to 20 hpf (Fig. S2). Very few Pax2a^+^ cells began to express low levels of dmKO2 reporter between 20 and 22 hpf (Fig. 2B). Thus, from 18 to 22 hpf, endodermal cells of the prospective thyroid region showed detectable FGF signaling limited to Pax2a^+^ thyroid progenitors but mostly lacked detectable BMP signaling activity. From 22 hpf onwards, we detected an increased number of endodermal cells with robust dmKO2 reporter expression and observed that this dmKO2 expression was largely restricted to Pax2a^+^ cells (Fig. 2B, Fig. S2). Moreover, when analyzing double-transgenic Tg(BRE:dmKO2),Et(*nkx2.4b*:YFP) embryos, we found that the onset of endodermal BMP signaling in individual cells coincided with induction of YFP expression in the same cell (Fig. 2D). When analyzing such double transgenic embryos throughout thyroid anlage formation (23 to 32 hpf), we could conclude that almost all dmKO2^+^ endodermal cells showed YFP expression and vice versa (see Fig. 2D).

In combination, the temporal mapping of signaling pathway activities identified FGF signaling as a defining characteristic of the early Pax2a^+^ thyroid progenitor cell population and revealed that the onset of BMP signaling in Pax2a^+^ cells is associated with the emergence of committed Pax2a^+^/Nkx2.4b^+^ thyroid precursors. Moreover, supporting the notion of cardiac mesoderm as an important source of FGF and BMP signals for thyroid specification (Vandernoot et al., 2020), we consistently observed the most intense biosensor reporter signals in endodermal cells located in close vicinity to the cardiac outflow tract (Fig. 2C,D).

### BMP and FGF signaling regulate the thyroid progenitor pool size

Our observations suggested that specific epistatic relationships of FGF signaling and Pax2a expression and of BMP signaling and Nkx2.4b expression might be at the core of the sequential induction of Pax2a^+^/Nkx2.4b^+^ thyroid precursors. We therefore used a small molecule inhibitor approach with a refined temporal treatment schedule in order to assess how periods sensitive to FGF and BMP inhibition relate to the temporal dynamics of endogenous FGF and BMP signaling.

**Fig. 3.**
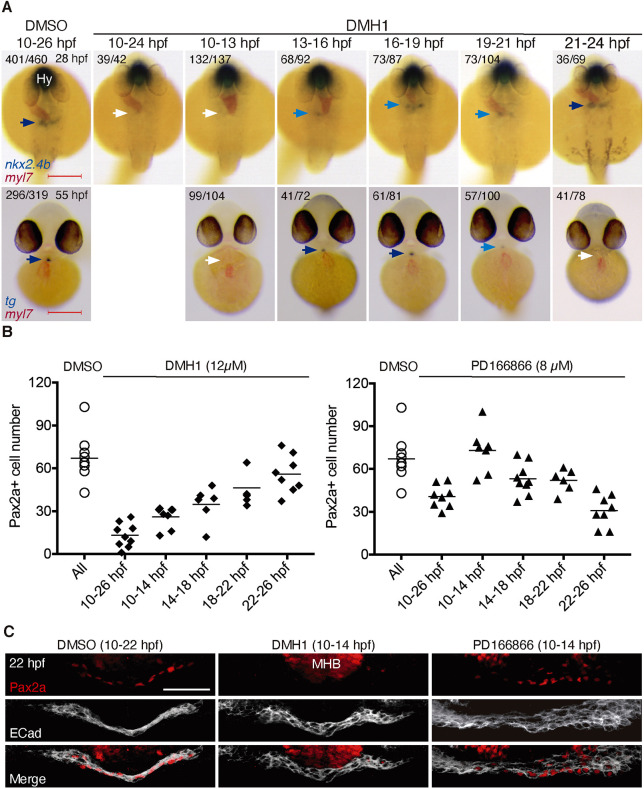
**Effects of timed inhibition of BMP and FGF signaling on thyroid specification.** (A) Major thyroid phenotypes recovered following DMH1 treatment during the indicated periods. Thyroid development was assessed by *in situ* hybridization of *nkx2.4b* at 28 hpf (dorsal views, anterior to the top) and *tg* at 55 hpf (ventral views, anterior to the top). Co-staining with *myl7* riboprobe shows localization of cardiac tissue. Numbers indicate the proportion of embryos with the represented phenotype out of the total number of observed embryos. Arrows point to thyroid tissue (colors highlight different levels of repression of thyroid marker expression). Hy, hypothalamus. Scale bars: 200 µm. (B) Quantification of the number of Pax2a^+^ cells in foregut endoderm of 28 hpf embryos following treatment with DMH1 or PD166866. Treatment intervals are indicated on the *x*-axis. DMSO (0.1%) treatment served as vehicle control group. Values obtained in individual embryos are shown and bars depict mean values for each experimental group. Values of the control group represent pooled control data from the various treatment periods. (C) Three-dimensional reconstruction of Pax2a expression domain in foregut of 22 hpf embryos treated with inhibitors of FGF (8 µM PD166866) or BMP signaling (12 µM DMH1) between 10 and 14 hpf. Confocal images of transverse sections are shown. E-cadherin (ECad) labels foregut epithelium. Note the complete loss of Pax2a expression in response to DMH1 treatment. Scale bar: 25 µm.

Treatment of embryos with potent small molecule inhibitors of BMP (12 µM DMH1) or FGF signaling (8 µM PD166866) throughout somitogenesis (10-24 hpf) completely prevented thyroid specification at 28 hpf based on *nkx2.4b* WISH analyses (Fig. 3A, Fig. S3). Short-term DMH1 treatment from 10 to 13 hpf was as effective as DMH1 treatment throughout somitogenesis in completely abolishing *nkx2.4b* expression at 28 hpf, whereas DMH1 treatments at later stages were generally less effective (Fig. 3A, Fig. S3). However, one remarkable finding was that a 2 h treatment (from 19 to 21 hpf) with DMH1 just before the onset of endogenous BMP signaling and *nkx2.4b* mRNA expression greatly reduced or abolished *nkx2.4b* expression in 28 hpf embryos and also had a lasting impact on *thyroglobulin* (*tg*) mRNA expression in 55 hpf embryos (Fig. 3A). In contrast, short-term PD166866 treatments during somitogenesis did not reveal a specific developmental period with heightened sensitivity of *nkx2.4b* mRNA expression to FGF inhibition.

We next analyzed the impact of timed treatments with DMH1 and PD166866 specifically on endodermal Pax2a expression (Fig. 3B). DMH1 treatment throughout somitogenesis (10-26 hpf) or during early somitogenesis (10-14 hpf) caused a dramatic reduction of Pax2a^+^ cells in foregut endoderm of 28 hpf embryos (Fig. 3B). Inhibition of BMP signaling at progressively later stages had a weaker effect on the number of Pax2a cells, indicating a crucial role of BMP signaling particularly during early somitogenesis for successful induction of a Pax2a^+^ progenitor cell population at later stages (Fig. 3C). Interestingly, short-term inhibition of FGF signaling revealed an inverse pattern as inhibitor treatment at early stages had no effect on Pax2a expression whereas treatment at late somitogenesis stages caused the strongest reduction of Pax2a^+^ cells (Fig. 3B).

Collectively, the temporal concordance of endodermal FGF signaling and the sensitivity of Pax2a expression to FGF activity inhibition is in support of an epistatic relationship of FGF signaling and Pax2a expression. The action of BMP signaling, however, appears to be more complex and involves regulation of both Pax2a and *nkx2.4b* expression during different developmental periods. Given that BMP signaling plays important roles in early mesoderm patterning and differentiation (Prummel et al., 2020; Row et al., 2018), we hypothesized that the suppressive effects of early global BMP inhibition might in part stem from impaired patterning of the foregut mesenchyme (including the cardiogenic LPM) rather than from direct effects on endoderm. To address the latter point, we treated embryos of the Tg(*dusp6*:d2EGFP) reporter line with DMH1 and analyzed FGF signaling in foregut endoderm of 22 hpf embryos (Fig. S4). Interestingly, global inhibition of BMP signaling during different somitogenesis stages not only reduced the number of Pax2a^+^/d2EGFP^+^ endodermal cells (Fig. S4A), but also resulted in an overall reduction of endodermal FGF signaling reporter expression (Fig. S4B).

### Overactivation of FGF signaling enlarges the pool of Pax2a^+^ thyroid progenitors

**Fig. 4.**
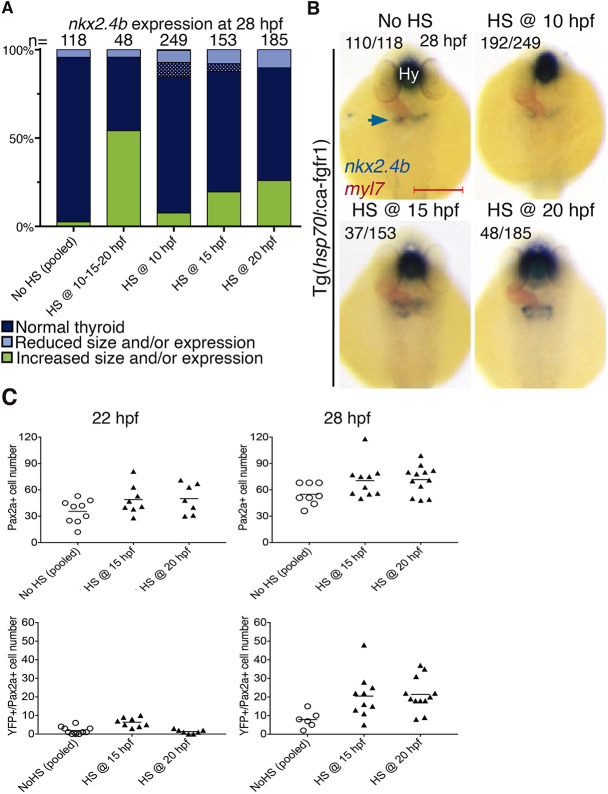
**Enhanced FGF signaling during late somitogenesis promotes thyroid precursor specification.** (A) Distribution of thyroid phenotypes (*nkx2.4b* expression at 28 hpf) induced by overactivation of FGF signaling using HS treatment of Tg(*hsp70l*:ca-fgfr1) embryos at the indicated time points. Phenotypic data shown include carriers and non-carriers of the HS-inducible transgene. Cases with abnormal morphology/positioning of *nkx2.4b* expression domain are highlighted by dotted texture overlays. (B) Thyroid phenotypes recovered at 28 hpf by *in situ* hybridization of *nkx2.4b* and *myl7* (cardiomyocytes) following overactivation of FGF signaling at the indicated time points. Arrow points to the thyroid anlage. Numbers indicate the proportion of embryos with the represented phenotype out of the total number of observed embryos and include carriers and non-carriers of the HS-inducible transgene. Dorsal views, anterior to the top. Hy, hypothalamus. Scale bar: 200 µm. (C) Total number of foregut endoderm cells expressing Pax2a (top) and cells expressing both Pax2a and YFP (bottom) following HS treatment of Tg(*hsp70l*:ca-fgfr1);Et(*nkx2.4b*:YFP) double-transgenic embryos at the indicated time points. Values for individual embryos and mean values (bars) are shown. Data shown include carriers and non-carriers of the HS-inducible transgene.

We next investigated if and when enhanced FGF signaling alters thyroid cell specification by using a transgenic zebrafish line, Tg(*hsp70*:ca-fgfr1), with a heat shock-inducible expression cassette for a constitutively active Fgfr1 (Marques et al., 2008). In these experiments, heterozygous Tg(*hsp70*:ca-fgfr1) founder fish were crossed with either WT or Et(*nkx2.4b*:YFP) fish so that 50% of the offspring would be carriers of the heat shock (HS)-inducible construct. HS induction of FGF signaling at 10 hpf did not affect *nkx2.4b* mRNA expression at 28 hpf, whereas HS treatment at later somitogenesis stages resulted in enhanced *nkx2.4b* expression in 28 hpf embryos (Fig. 4A,B). Although an enlarged *nkx2.4b* expression domain was observed in only 20-25% of heat-shocked embryos, subsequent genotyping of a portion of stained embryos confirmed that all of the embryos presenting enhanced *nkx2.4b* staining were indeed carriers of the HS-inducible transgene.

IF analyses of heat-shocked embryos showed that HS treatment at 15 and 20 hpf increased the number of Pax2a^+^ endodermal cells in several embryos analyzed at 22 and 28 hpf (Fig. 4C). Although firm mounting of tissue sections on glass slides prevented PCR-based genotyping, we nevertheless regard the increased number of Pax2a cells in a subset of heat-shocked embryos as an indication that overactivation of FGF signaling promotes an increase in the number of Pax2a^+^ thyroid progenitors above the range observed in controls.

When performing a similar analysis in double transgenic Tg(*hsp70*:ca-fgfr1);Et(*nkx2.4b*:YFP) embryos, we observed that HS treatment at 15 and 20 hpf increased the number of YFP^+^ endodermal cells in many embryos analyzed at 28 hpf (Fig. 4C). We also noted that HS-induced overactivation of FGF signaling did not evoke a precocious onset of YFP expression as heat-shocked embryos still lacked YFP expression at 20/21 hpf (data not shown) and numbers of endodermal YFP^+^ cells in 22 hpf embryos were still low, similar to stage-matched controls (Fig. 4C).

### Overactivation of BMP signaling in early somitogenesis induces supernumerary thyroid precursors

We next used a similar methodology to analyze if and when overactivation of BMP signaling could affect thyroid specification. For this purpose, we crossed heterozygous Tg(*hsp70*:*bmp2b*) founder fish carrying an HS-inducible expression cassette for zebrafish Bmp2b (Chocron et al., 2007) with either WT or Et(*nkx2.4b*:YFP) fish and examined the offspring following timed HS treatment. Global overactivation of BMP signaling at 10 hpf resulted in a ventralized body morphology phenotype (Chocron et al., 2007; Kishimoto et al., 1997) in about half of all heat-shocked embryos. This ventralized phenotype was specific for HS treatments performed at 10 hpf and greatly facilitated the identification of embryos affected by early BMP overactivation.

**Fig. 5.**
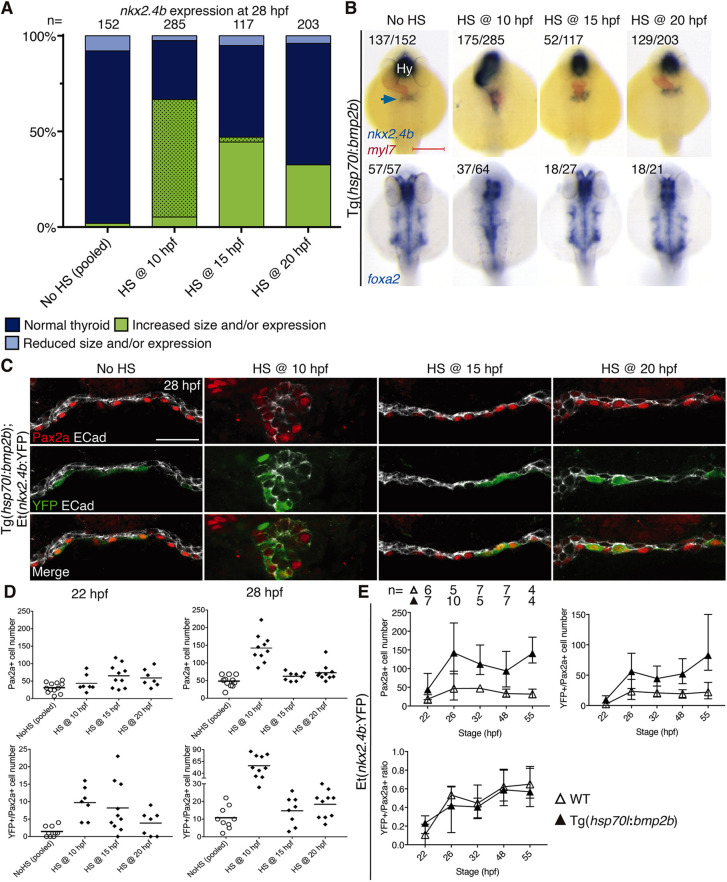
**Enhanced BMP signaling promotes thyroid precursor specification.** (A) Distribution of thyroid phenotypes (*nkx2.4b* expression at 28 hpf) induced by overactivation of BMP signaling using HS treatment of Tg(*hsp70l*:*bmp2b*) embryos at the indicated time points. Phenotypic data shown include carriers and non-carriers of the HS-inducible transgene. Cases with abnormal morphology/positioning of *nkx2.4b* expression domain are highlighted by dotted overlays. (B) Thyroid and endodermal phenotypes recovered at 28 hpf by *in situ* hybridization of *nkx2.4b*, *myl7* (cardiomyocytes) and *foxa2* (foregut endoderm) following overactivation of BMP signaling at the indicated time points. Numbers indicate the proportion of embryos with the phenotype shown out of the total number of embryos observed and include carriers and non-carriers of the HS-inducible transgene. Arrow points to the thyroid anlage. Dorsal views, anterior to the top. Hy, hypothalamus. Scale bar: 200 µm. (C) Pax2a, YFP and E-cadherin (ECad) expression in foregut endoderm of Tg(*hsp70l*:*bmp2b*);Et(*nkx2.4b*:YFP) double-transgenic embryos at 28 hpf. Confocal images of transverse sections are shown. Note the severely perturbed morphology (rod-like) of the foregut following HS treatment at 10 hpf. Scale bar: 25 µm. (D) Total number of foregut cells expressing Pax2a (top) and cells expressing both Pax2a and YFP (bottom) following HS treatment of Tg(*hsp70l*:*bmp2b*);Et(*nkx2.4b*:YFP) double-transgenic embryos at the indicated time points. Values for individual embryos and mean values (bars) are shown. Data for HS at 10 hpf are from ventralized embryos (carriers of the HS transgene). Data for HS treatments at 15 and 20 hpf include carriers and non-carriers of the HS-inducible transgene. (E) Temporal analyses of thyroid markers in Tg(*hsp70l*:*bmp2b*);Et(*nkx2.4b*:YFP) embryos heat-shocked at 10 hpf. Carriers of the HS transgene were identified based on their unique ventralized body morphology. Total number of foregut cells expressing Pax2a (top left) and cells expressing both Pax2a and YFP (top right) are shown for the indicated time points. Bottom graph shows the percentage of Pax2a cells co-expressing YPF. Mean±s.d. is shown.

Formation of the thyroid anlage in ventralized embryos was severely perturbed as judged from the size, shape and location of the *nkx2.4b* expression domain at 28 hpf. The thyroid anlage was greatly enlarged and showed a caudal expansion along the midline, flanked bilaterally by elongated strands of *myl7*-expressing cardiac mesoderm (Fig. 5B, Fig. S5). Expression of the foregut endoderm marker *foxa2* (Fig. 5B) and GFP reporter in embryos of the Tg(*sox17*:EGFP) endodermal reporter line (Fig. S5B) revealed severe malformations of the pharyngeal endoderm in response to early overactivation of BMP signaling.

Confocal imaging also showed that, instead of a sheet-like morphology, the foregut endoderm of heat-shocked Tg(*hsp70*:*bmp2b*);Et(*nkx2.4b*:YFP) embryos presented a rod-like morphology (Fig. 5C). Both Pax2a^+^/YFP^−^ progenitors and Pax2a^+^/YFP^+^ thyroid precursors were intermingled along the dorsoventral axis and over long stretches along the AP axis of these rod-like endodermal structures (Fig. 5C). In 28 hpf embryos displaying a ventralized phenotype, quantitative analyses estimated 3- to 5-fold increases in the number of Pax2a^+^/YFP^−^ and Pax2a^+^/YFP^+^ cells, respectively (Fig. 5D).

Despite the detection of a 6-fold increase in number of Pax2a^+^/YFP^+^ cells in 22 hpf embryos after early BMP overactivation (Fig. 5D), the absence of precocious YFP expression in 19/20 hpf embryos (data not shown) indicate that there was no dramatic change in the onset of Nkx2.4b induction. Temporal analyses of ventralized embryos showed that the specification of a supernumerary thyroid precursor cell population yields an enlarged thyroid primordium in 55 hpf embryos containing a 3-fold higher number of thyroid cells compared with controls (Fig. 5E, Fig. S5C).

### Overactivation of BMP signaling in late somitogenesis accelerates thyroid precursor specification

Global overactivation of BMP signaling at later somitogenesis stages did not result in foregut malformations (Fig. 5B), but still caused enhanced *nkx2.4b* expression at 28 hpf (Fig. 5A,B). PCR genotyping of a subset of stained specimens confirmed that embryos with enhanced *nkx2.4b* staining carried the HS-inducible transgene. Additional IF analyses of heat-shocked embryos showed a shift towards higher numbers of Pax2a^+^ foregut cells (above the range seen in controls) at 22 hpf (Fig. 5D). Although the firm tissue mounting prevented us from using PCR genotyping to directly link increased Pax2a cell numbers to the presence of the HS construct, the detection of increased Pax2a cell numbers (above the range detected in controls) in about half of all heat-shocked embryos analyzed at 22 hpf strongly indicates that BMP overactivation at 15 or 20 hpf induced a surplus of Pax2a^+^ thyroid progenitors at this stage. The stimulatory effect of BMP signaling on Pax2a cell numbers appeared to level off at 28 hpf (the stage when total Pax2a cell numbers peak in normal development) as heat-shocked embryos showed, if any, only small increases in Pax2a cell numbers (Fig. 5D). In the absence of more definitive genotyping data, we cautiously argue that enhanced BMP signaling at later somitogenesis stages accelerates the initial induction of Pax2a in the foregut (at 22 hpf) without strongly affecting the final size of the thyroid progenitor pool (at 28 hpf).

Overactivation of BMP signaling at 15 and 20 hpf strongly accelerated the differentiation of Pax2a^+^/Nkx2.4b^+^ thyroid precursors at 22 hpf compared with controls (Fig. 5D). By 28 hpf, the number of Pax2a^+^/Nkx2.4b^+^ thyroid precursors was moderately increased in a subset of heat-shocked embryos, mirroring the moderate increase in *nkx2.4b* mRNA staining seen in genotyped transgene carriers at 28 hpf (Fig. 5B). However, we also observed that enhanced BMP signaling failed to induce YFP in all Pax2a^+^ cells (see Fig. 5C) so that Nkx2.4b expression was still restricted to only a fraction of endodermal Pax2a^+^ cells. Collectively, these experiments show that ectopic overactivation of BMP signaling has complex consequences for foregut morphogenesis and thyroid specification depending on the developmental stage and include effects on both Pax2a and Nkx2.4b expression dynamics.

### Cooperative FGF and BMP signaling is required for thyroid specification

**Fig. 6.**
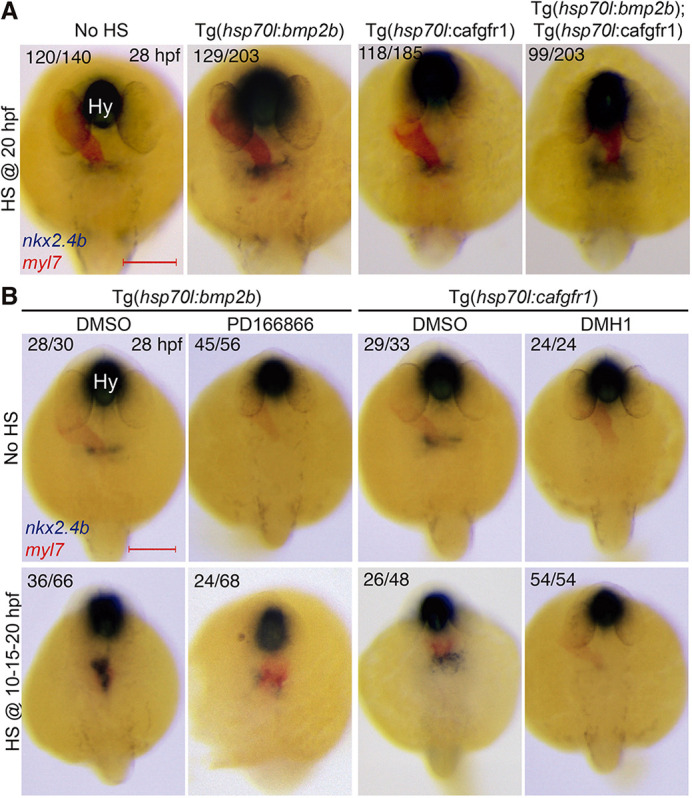
**Cooperative effects of FGF and BMP on thyroid specification.** (A) Representative thyroid phenotypes recovered at 28 hpf by *in situ* hybridization of *nkx2.4b* and *myl7* (cardiomyocytes) following HS treatment of double-transgenic Tg(*hsp70l*:*bmp2b;hsp70l*:ca-fgfr1) embryos at 20 hpf. Carriers of the individual transgenes were identified by genotyping. Note the expansion of the thyroidal *nkx2.4b* expression domain in embryos with concurrent overactivation of both pathways. (B) Top: Thyroid phenotypes recovered at 28 hpf after inhibition of FGF (8 µM PD166866) and BMP signaling (12 µM DMH1) from 10 to 24 hpf. Bottom: Rescue efficacy by concurrent overactivation of BMP or FGF signaling (repeated HS at 10, 15 and 20 hpf). Note that overactivation of BMP signaling could partially restore *nkx2.4b* expression in embryos treated with FGF inhibitor. Numbers indicate the proportion of embryos with the represented phenotype out of the total number of observed embryos and include carriers and non-carriers of the HS-inducible transgene. Dorsal views, anterior to the top. Hy, hypothalamus. Scale bars: 200 µm.

Our manipulation of individual pathways identified unique and overlapping roles of FGF and BMP signaling with respect to Pax2a and Nkx2.4b expression. We next generated Tg(*hsp70*:ca-fgfr1;*hsp70*:*bmp2b*) double-transgenic embryos and performed HS experiments to assess the combined effect of FGF and BMP overactivation on thyroid anlage formation. We focused our analyses on effects caused by HS treatment at 20 hpf so that pathway overactivation would cover the period of both Pax2a and Nkx2.4b induction. Heat-shocked embryos were analyzed at 28 hpf for *nkx2.4b* mRNA expression and genotyped by PCR after staining. Examination of embryos carrying the different overexpression constructs (see Fig. 6A) showed that overactivation of BMP or FGF alone resulted in enhanced *nkx2.4b* mRNA expression, as seen in previous experiments, whereas concurrent overactivation of both signaling pathways caused a much stronger increase in *nkx2.4b* staining intensity and an overall expansion of the thyroid anlage (Fig. 6A). These findings suggests that cooperative action of FGF and BMP signaling at late somitogenesis regulates thyroid specification.

To further bolster this contention, we also assessed whether the loss of one pathway could be compensated for by overactivation of the other (Fig. 6B). For this purpose, we repeatedly heat-shocked Tg(*hsp70l*:*bmp2b*) embryos throughout somitogenesis and raised these embryos in the presence or absence of the FGF inhibitor PD166866 (8 µM) from 10 to 28 hpf. In these experiments, FGF inhibitor treatment completely abolished *nkx2.4b* mRNA expression in 28 hpf embryos and BMP overactivation in inhibitor-treated embryos could only partially rescue *nkx2.4b* mRNA expression, but failed to induce the marked thyroid anlage expansion seen in vehicle-treated embryos. We also observed that repeated HS of Tg(*hsp70l*:ca-fgfr1) embryos could not rescue the failure of thyroid anlage formation caused by treatment of embryos from 10 to 28 hpf with the BMP inhibitor DMH1 (Fig. 6B). Collectively, these data are consistent with a model in which FGF signaling has permissive whereas BMP signaling has superordinate inductive capacity to initiate *nkx2.4b* expression in the foregut endoderm.

### Pax8 and Nkx2-1 expression during early mouse foregut morphogenesis

**Fig. 7.**
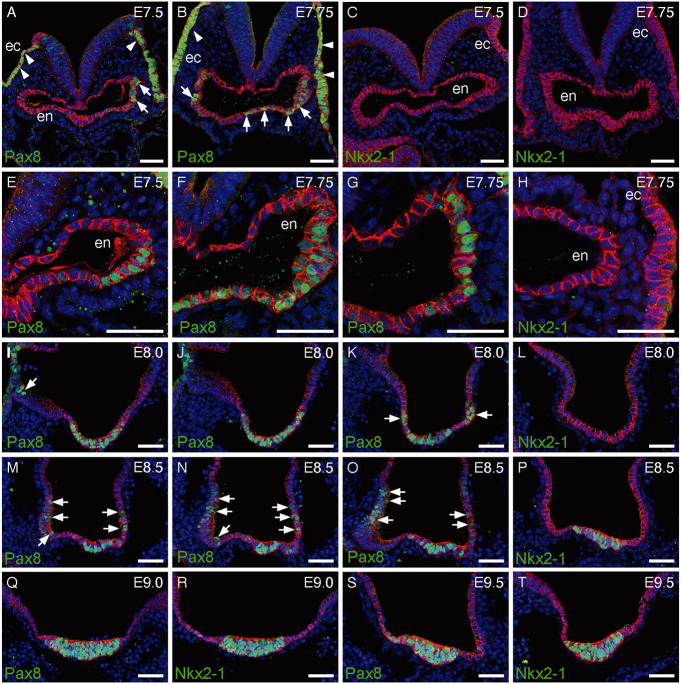
**Pax8 and Nkx2-1 expression during mouse foregut morphogenesis.** (A-D) IF staining of E7.5 and E7.75 embryos at the level of the forming foregut pocket shows strong Pax8 expression in the non-neuronal, ectodermal epithelium (ec, arrowheads) and in epithelial cells of the foregut endoderm (en, arrows). These embryos had a TS12 morphology and Nkx2-1 expression was not detectable at this stage. E-cadherin (cadherin 1) staining (red) is used to label epithelial structures in all panels. (E-H) Higher magnification views of foregut endoderm in E7.5-E7.75 embryos illustrate variable levels of Pax8 staining in endodermal cells (F). Confocal *z*-sections acquired at 50 µm intervals along the AP axis show anterior Pax8 expression (F) in both ventral and lateral foregut endoderm whereas posterior Pax8 expression (G) is limited to lateral endoderm. Note the complete absence of Nkx2-1 expression (H). (I-K) Confocal *z*-sections acquired at 30 µm intervals along the AP axis of E8.0 embryo foregut illustrate how the broad Pax8 expression domain in anterior ventral foregut endoderm (I) expands to gradually include more lateral domains at posterior locations (arrows in K). This embryo had a TS13 morphology. (L) Nkx2-1 expression was not detectable in E8.0 embryos. (M-P) Upon completion of embryo turning at E8.5, expression of both Pax8 (M-O) and Nkx2-1 (P) is detectable in ventral foregut endoderm of TS14 embryos. Confocal *z*-sections acquired at different levels along the AP axis show widespread persistence of Pax8 expression in the lateral foregut epithelium (arrows in M-O). (Q-T) Co-expression of Pax8 and Nkx2-1 in the morphologically distinct, ventral midline primordium of the thyroid of E9.0 (Q,R) and E9.5 (S,T) embryos. Scale bars: 50 µm.

In order to understand if a similar sequence of Pax8 and Nkx2-1 expression exists during mammalian foregut morphogenesis, we additionally collected mouse embryos from the eighth to the tenth day of pregnancy and performed confocal microscopy of IF-stained vibratome sections of the anterior foregut region. The earliest stage analyzed comprised mouse embryos collected at E7.5 showing Theiler stage (TS) 12 morphology and formation of a rudimentary foregut pocket (Fig. S6). Confocal microscopy revealed strong Pax8 staining of non-neuronal ectodermal epithelium and, in addition, a weaker but specific Pax8 staining of small clusters of endodermal epithelial cells (Fig. 7A,B). Patches of Pax8^+^ cells were initially limited to lateral regions of the foregut pocket (Fig. 7E), but as morphogenesis progressed Pax8^+^ cells were frequently found in more medial regions of the ventral foregut epithelium (Fig. 7F) and the intensity of the Pax8 IF staining gradually increased over time. We also noted that ventral Pax8 expression was seen in rostral regions (Fig. 7F), whereas in more caudal positions, Pax8 expression was limited to lateral foregut epithelium (Fig. 7G). Notably, we never observed Pax8^+^ cells in the dorsal foregut epithelium at any stage analyzed in this study. IF staining for Nkx2-1 did not show any detectable Nkx2-1 expression in either endodermal or ectodermal epithelia of TS12 embryos (Fig. 7C,D,H).

In TS13 embryos collected at E8.0, we observed a large domain of supernumerary, endodermal Pax8^+^ cells but Nkx2-1 expression was still not detectable (Fig. 7I-L, Fig. S6). Confocal images shown in Fig. 7I-L were acquired at 30 µm intervals to illustrate the large anterior–posterior expansion of the Pax8 expression domain in TS13 embryos. We also noted that the Pax8 expression domain became more continuous in TS13 embryos and contained the ventral midline foregut epithelium at anterior positions while expanding to more lateral endodermal epithelial cells at caudal positions (see Fig. S6).

Expression of Nkx2-1 in the foregut endoderm was first observed in TS14 embryos collected at E8.5. The expression domain of Nkx2-1 was strictly limited to the ventral midline foregut epithelium (Fig. 7P). Notably, Pax8 IF analysis of TS14 embryos from the same litter showed Pax8 expression not only in the ventral epithelium but still in the lateral wall of the foregut along a large stretch of the AP axis (Fig. 7M-O). Although specifications of antibodies used in this study did not allow for direct co-staining of tissues with Pax8 and Nkx2-1 antibodies, the individual staining patterns of Pax8 and Nkx2-1 in E9.0 and E9.5 embryos strongly indicate that cells of the ventral midline thyroid primordium co-express both transcription factors (Fig. 7R-T). We further noted that Pax8 expression had ceased in endodermal cells outside the midline primordium by E9.0. Collectively, our analyses of Pax8 and Nkx2-1 expression in mouse embryos revealed an evolutionarily conserved temporal pattern with the onset of Pax8/Pax2a expression preceding Nkx2-1/Nkx2.4b expression.

## DISCUSSION

**Fig. 8.**
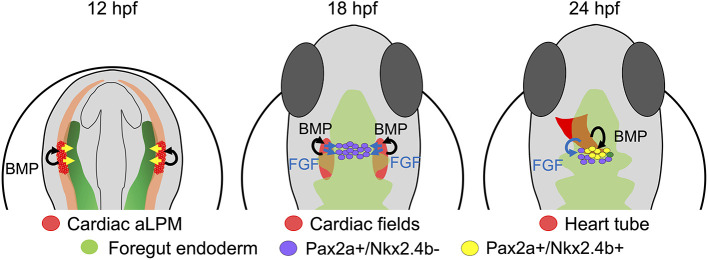
**Refined model of thyroid precursor specification.** During early somitogenesis, the anterior foregut endoderm (green) receives patterning signals (yellow arrows) from the anterior lateral plate mesoderm (aLPM). At around 18 hpf, FGF signals (blue arrows) derived from the adjacent cardiac mesoderm (red) induce Pax2a expression in endodermal cells. Up to this stage, active BMP signaling is limited to mesodermal cells. Around 23/24 hpf, a subpopulation of Pax2a^+^ endodermal cells, located closest to the cardiac outflow tract mesoderm, receives BMP signals inducing Nkx2.4b expression. Pax2a^+^/Nkx2.4b^+^ cells represent committed thyroid cell precursors, whereas Pax2a^+^/Nkx2.4b^−^ cells assume alternative cell fates.

Current models of thyroid development hold that a concurrent induction of PAX8 and NKX2-1, or their teleost functional paralogs Pax2a and Nkx2.4b, is the initiating event of thyroid specification (De Felice and Di Lauro, 2011; Nilsson and Fagman, 2017; Porazzi et al., 2009; Szinnai, 2014). In this study, we generated novel insights into the developmental dynamics of zebrafish thyroid cell specification, permitting us to formulate a refined model of early thyroid cell differentiation (Fig. 8).

First, we observed that the thyroid transcription factors Pax2a and Nkx2.4b are sequentially induced in the anterior foregut endoderm. Specifically, we identified a population of Pax2a^+^ endodermal cells that emerges several hours before the onset of Nkx2.4b expression in the thyroid anlage region. We describe these early Pax2a^+^ cells as endodermal thyroid progenitors to distinguish them from subsequently evolving Pax2a^+^/Nkx2.4b^+^ cells, which we refer to as lineage-committed thyroid precursors in accordance with previously proposed terminology (De Felice and Di Lauro, 2011). The existence of an equivalent endodermal progenitor cell population expressing Pax8 prior to the onset of Nkx2-1 had not been previously reported in any of the commonly used model species. However, our analyses of early mouse embryos confirmed that a similar supernumerary pool of Pax8^+^ endodermal cells exists during mammalian embryonic foregut development. Although more studies in other species are needed, these findings lead us to predict that the molecular mechanisms of zebrafish thyroid specification reported here are likely conserved among vertebrates.

Our data indicate that thyroid lineage commitment is a multistep process whereby patterning processes initially yield a spatially restricted pool of Pax2a^+^ cells within the anterior foregut endoderm and a subsequent differentiation event results in the emergence of Pax2a^+^/Nkx2.4b^+^ thyroid precursors from this pool. This model is supported not only by the earlier appearance of Pax2a relative to Nkx2.4b expression, but also by our observation that Nkx2.4b induction occurs exclusively in Pax2a^+^ endodermal cells. Another key finding of our study was that the number of Pax2a^+^ cells populating the prospective thyroid field exceeds the final number of thyroid precursors contained in the thyroid bud. Throughout all stages of thyroid anlage formation (from 23/24 to 28 hpf), a variable number of Pax2a^+^ cells did not co-express Nkx2.4b and our temporal analyses suggest that these Pax2a^+^/Nkx2.4b^−^ cells do not contribute to the thyroid primordium at later stages. We therefore conclude that Pax2a expression alone does not determine thyroid cell fate in zebrafish, but that thyroid lineage commitment requires co-expression of Pax2a and Nkx2.4b. Over time, the initially supernumerary Pax2a^+^ cell population diminishes in size and, currently, we cannot rule out apoptotic removal of Pax2a^+^/Nkx2.4b^−^ cells as this hypothesis has not been experimentally addressed so far. However, given that endodermal cells with gradually decreasing Pax2a^+^ immunolabeling remained well integrated in the ventral foregut epithelium, we currently rather favor a model in which, in the absence of Nkx2.4b expression, Pax2a^+^ cells assume alternative cell fates within the pharyngeal epithelium after downregulating Pax2a expression.

According to this concept, multiple mechanisms might regulate endodermal Pax2a expression during early foregut development. Early stimulatory signals, likely related to AP foregut patterning, induce Pax2a expression in a regionally defined, medio-lateral stripe of the pharyngeal endoderm, but these promoting effects appear to be transient. Additional regulatory mechanisms, linked to the co-expression of Nkx2.4b, are required to stabilize Pax2a expression specifically in the thyroid cell lineage. Such a model of Pax2a regulation is in accordance with phenotypic studies of Nkx2-1/Nkx2.4b-deficient mouse and zebrafish embryos showing that early *Pax8*/*pax2a* expression in the thyroid region is unaffected in Nkx2-1/Nkx2.4b-deficient embryos, but *Pax8*/*pax2a* expression is subsequently lost in the absence of *Nkx2-1*/*nkx2.4b* function (Elsalini et al., 2003; Parlato et al., 2004).

Although our current study showed that Nkx2.4b expression occurs exclusively in Pax2a^+^ endodermal cells, Pax2a expression itself is dispensable for Nkx2.4b induction in the zebrafish thyroid anlage (Trubiroha et al., 2018), as is Pax8 expression for Nkx2-1 induction in the murine thyroid anlage (Parlato et al., 2004). Thus, although Pax2a expression marks foregut endodermal cells that are competent to induce Nkx2.4b expression, absence of Pax2a expression does not impair this competence. Pax2a is, however, strictly required to maintain the thyroid fate and to promote further thyroid differentiation (Porreca et al., 2012; Trubiroha et al., 2018; Wendl et al., 2002).

The identification of distinct cell states during thyroid specification provided us with an opportunity to examine the role of crucial extrinsic factors, such as FGF and BMP signaling, in the spatiotemporal regulation of these processes. Prior to the appearance of Pax2a-expressing cells at 18 hpf, the prospective thyroid endoderm lacks detectable FGF and BMP signaling activity, an observation that aligns well with reported posteriorizing activities of FGF and BMP on endoderm patterning (Green et al., 2011; Tiso et al., 2002). According to our refined model, FGF signaling within the foregut endoderm is required for the early onset of Pax2a expression. This contention is supported by several observations. First, Pax2a^+^ cells of the foregut endoderm are characterized by enhanced intracellular FGF signaling, whereas adjacent Pax2a-negative endoderm lacks such FGF signaling activity. Second, blockade of FGF signaling diminishes the number of Pax2a^+^ cells in the thyroid field, whereas late somitogenesis overactivation of FGF signaling causes induction of Pax2a expression in an increased number of endodermal cells. Finally, the proposed direct FGF regulation of early Pax2a expression in zebrafish foregut endoderm mirrors the capacity of FGF2 to induce high levels of PAX8 expression in human anterior foregut endoderm (Dye et al., 2015).

However, globally enhanced FGF signaling did not cause ectopic patches of Pax2a expression outside the thyroid field region in zebrafish embryos, indicating that FGF alone has not sufficient instructive activity to convert neighboring endoderm into progenitors of the thyroid cell lineage. This conclusion is in accordance with a lack of ectopic thyroid specification after grafting of FGF-soaked beads in the foregut region of zebrafish embryos (Wendl et al., 2007). It remains currently unclear whether the spatial restriction of FGF effects on Pax2a expression is due to a cellular competence set by early endoderm patterning cues or if FGF signaling acts permissively together with other signaling cues, including BMP-dependent pathways (Haerlingen et al., 2019; Kurmann et al., 2015; Porazzi et al., 2012; Porreca et al., 2012; Rurale et al., 2020; Serra et al., 2017; Wendl et al., 2007).

The intimate association between onset of BMP signaling and concurrent upregulation of *nkx2.4b* expression in a subset of Pax2a^+^ cells clearly positions BMP activity as a key signaling cue for the second step towards thyroid cell lineage commitment. However, our experimental data also revealed that BMP signaling plays more complex roles during thyroid specification, including the timely regulation of endodermal Pax2a induction as well as other complex endoderm-mesoderm interactions shaping the normal development of the foregut region.

BMP signaling is active in the anterior LPM near the thyroid-forming endoderm and is required for normal differentiation of these mesodermal derivatives (de Pater et al., 2012; Prummel et al., 2019). BMP-mediated patterning of mesoderm development is therefore likely involved in the unique anatomical constellation resulting from early overactivation of BMP signaling. Here, the dramatic caudal expansion of the thyroid anlage was closely associated with the presence of caudally elongated strands of cardiac mesoderm bilaterally flanking a dysmorphic endoderm. Assuming cardiac mesoderm as a major source of FGF and BMP signals instructing thyroid specification at late somitogenesis, the latter phenotype serves as an example for the complex direct and indirect action of BMP during mesoderm patterning and endodermal thyroid induction. Although our observations provide further support for the role of cardiac mesoderm-derived signaling for early thyroid differentiation (Haerlingen et al., 2019; Vandernoot et al., 2020; Wendl et al., 2007), dissection of precise signaling mechanisms will be a formidable challenge for future studies given the multitude of FGFs and BMPs that are expressed in precardiac and cardiac mesoderm (Chocron et al., 2007; Chung et al., 2008; Longmire et al., 2012; Mesbah et al., 2012; Romitti et al., 2022; Thisse et al., 2004).

By integrating the various experimental findings, we postulate a refined model of thyroid specification in that the midline positioning of the thyroid anlage stems from the intersection of two regional specification events (see Fig. 8). Spatial coordinates for thyroid specification along the AP axis would be determined by anterior LPM-derived patterning cues (including FGF and BMP ligands) resulting in a foregut endoderm region with a medial-to-lateral distribution of Pax2a^+^ cells at a defined AP level. This field of Pax2a^+^ cells has a competence for BMP-induced Nkx2.4b expression, but only the Pax2a^+^ cells the closest to the cardiac mesoderm forming the arterial pole of the heart tube might receive sufficiently strong BMP stimulation for Nkx2.4b induction owing to their proximity. Thus, the midline positioning of the arterial pole of the heart tube would provide spatial coordinates along the medial-to-lateral axis to restrict thyroid precursor specification to more medial positions within the broader Pax2a^+^ field. Finally, the proposed step-wise specification process provides a novel concept that could aid our understanding of the various thyroid phenotypes arising in response to cardiac maldevelopment (Olivieri et al., 2002; Opitz et al., 2012; Haerlingen et al., 2019; Vandernoot et al., 2020).

## MATERIALS AND METHODS

### Zebrafish

Zebrafish husbandry and experiments with embryos were performed under standard conditions as per the Federation of European Laboratory Animal Science Associations (FELASA) guidelines, and in accordance with institutional (Université Libre de Bruxelles, ULB) and national ethical and animal welfare guidelines and regulation, which were approved by the ethical committee for animal welfare (CEBEA) from the Université Libre de Bruxelles (protocols 578N-579N).

Zebrafish (*Danio rerio*) embryos were collected within 30 min after spawning, raised in fish water supplemented with 0.01 mg/l methylene blue (referred to as embryo medium) at 28.5°C and staged as described (Haerlingen et al., 2019; Kimmel et al., 1995). Media were supplemented with 0.003% 1-phenyl-2-thiourea (PTU) from 24 hpf onwards to prevent pigmentation. Embryos were enzymatically dechorionated by incubation in embryo medium containing 0.6 mg/ml pronase at room temperature (Haerlingen et al., 2019). For live analyses and before sampling or fixation, embryos were anesthetized in medium containing 0.02% tricaine. Embryos were fixed in 4% paraformaldehyde (PFA) solution in PBS overnight at 4°C with gentle agitation.

The following zebrafish lines were used: pigmentless *casper* strain (White et al., 2008), Et(*nkx2.4b*:YFP) (CLGY576) (Ellingsen et al., 2005), Tg(*hsp70*:ca-fgfr1,*cryaa*:DsRed)^pd1^ abbreviated as Tg(*hsp70*:ca-fgfr1) (Marques et al., 2008), Tg(*hsp70*:*bmp2b*)^fr13^ (Chocron et al., 2007), Tg(-5.0*sox17*:EGFP)^ha01^ abbreviated as Tg(*sox17:*EGFP) (Mizoguchi et al., 2008), Tg(*myl7*:EGFP)^twu26^ (Huang et al., 2003), Tg(*dusp6*:d2EGFP)^pt6^ (Molina et al., 2007), Tg(BRE-AAVmlp:dmKO2)^mw40^ abbreviated as Tg(BRE:dmKO2) (Collery and Link, 2011).

### Mouse embryos

Adult pregnant mice (C57BL/6J background) were provided by the animal facility of the Charité Universitätsmedizin Berlin and kept under standard conditions (12 h light/dark cycle) with *ad libitum* access to food and water. The morning the copulation plug was identified was defined as E0.5 and embryos were collected between E7.5 and E9.5. Immediately after sacrificing the pregnant animal by CO_2_ inhalation and cervical dislocation, embryos were dissected intact in ice-cold PBS and fixed in 4% PFA for 24 h at 4°C. Experiments involving animals were conducted in compliance with the institution's ethical guidelines and performed in accordance to ARRIVE guidelines, the European Union Directive 2010/63/EU and the German guideline for care and use of laboratory animals after approval by institutional authorities.

### Small molecule treatment

Stock solutions of 20 mM PD166866 (Sigma-Aldrich, PZ0114) and 10 mM DMH1 (Sigma-Aldrich, D8946) were prepared in DMSO and aliquots were stored at −20°C until use. Treatment solutions were freshly prepared by diluting stock solutions in embryo medium and kept at 28.5°C. The final test concentrations of 8 µM PD166866 and 12 µM DMH1 were selected based on previous studies (Haerlingen et al., 2019). Embryos treated with 0.1% DMSO served as a vehicle control group for all small molecule treatment experiments. Media were supplemented with 0.01 mg/ml Methylene Blue to prevent bacterial growth and 0.003% PTU to prevent pigmentation.

Two hours before treatment initiation, embryos were staged and stage-matched embryos were pooled and randomly allocated to the different experimental treatments into 60 mm Petri dishes (40-50 maximum per dish). Treatment periods are indicated in the text and in the corresponding figures. All treatments were performed in duplicate. All incubations of embryos in small molecule solutions were performed at 28.5°C in the dark. After completion of small molecule treatment, embryos were washed three times with fresh embryo medium before transfer into clean 96 mm dishes filled with fresh embryo medium.

### HS treatment

Embryos for HS experiments were obtained by crossing heterozygotic founder fish of the Tg(*hsp70l*:ca-fgfr1) or Tg(*hsp70l*:*bmp2b*) lines with homozygotic founder fish of the indicated reporter lines Et(*nkx2.4b*:YFP), Tg(*myl7*:EGFP) and Tg(*sox17*:EGPF). Stage-matched embryos obtained from such crosses were pooled and randomly allocated to the differentially timed HS experimental groups. HS was typically applied at 10 hpf (early somitogenesis), 15 hpf (mid somitogenesis) or 20 hpf (late somitogenesis). Some experiments involved repeated HS treatment of embryos at 10, 15 and 20 hpf. HS experiments were performed at least in duplicate. For HS treatment, embryo medium was entirely removed and replaced by pre-warmed embryo medium (40°C) and embryos were incubated at 40°C for 30 min. After the HS period, the warm medium was removed and replaced by fresh embryo medium and embryos were further raised under standard conditions at 28.5°C. In some experiments, HS treatments were combined with small molecule inhibitor treatments. Here, pre-warmed embryo medium containing the indicated small molecule inhibitors was used for the 40°C incubation to ensure continuous exposure to inhibitors.

### Single- and dual-color WISH

DNA templates for synthesis of *nkx2.4b* (previously *nkx2.1a*, ZDB-GENE-000830-1), *tg* (ZDB-GENE-030519-1), *hhex* (ZDB-GENE-980526-299), *pax2a* (ZDB-GENE-990415-8) and *foxa2* (ZDB-GENE-980526-404) riboprobes were generated by PCR essentially as described (Opitz et al., 2011). Riboprobes labeled with digoxigenin (DIG), fluorescein (FLU), or dinitrophenol (DNP) were prepared as described (Opitz et al., 2011) using DIG RNA labeling kit (Roche), FLU RNA labeling kit (Roche) or DNP-UTP (PerkinElmer).

The WISH protocol was based on that of Thisse and Thisse (2008) with modifications as described (Haerlingen et al., 2019; Opitz et al., 2011). Briefly, PFA-fixed embryos were washed three times for 10 min with PBS containing 0.1% Tween 20 (PBST) followed by gradual dehydration through a series of methanol/PBST solutions (25, 50, 75, 100%) and stored in 100% methanol at −20°C for at least 24 h. Following gradual rehydration in PBST, embryos were permeabilized by short-term proteinase K treatment, post-fixed for 20 min in 4% PFA and rinsed in PBST. Hybridization of embryos was performed at 65°C essentially as described (Opitz et al., 2011).

Single-color staining of embryos hybridized with DIG-labeled riboprobes was performed using anti-DIG antibody conjugated to alkaline phosphatase (AP) (1:6000; 11093274910, Roche) with BM Purple (Roche) as AP substrate as described (Opitz et al., 2011). Dual-color staining was performed as described (Haerlingen et al., 2019). Briefly, following hybridization of embryos with multiple differentially labeled riboprobes, we first detected the DIG-labeled *nkx2.4b* riboprobe with anti-DIG-AP antibody (1:6000) and BM Purple staining and the DNP-labeled *tg* riboprobe with anti-DNP antibody (MB-3100, Vector Laboratories, 1:500) and NBT/BCIP (Roche) staining. Stained specimens were washed in PBST, quickly cleared in 100% methanol to remove unspecific background staining, rehydrated in PBST, and incubated in 1% (w/v) glycine solution adjusted to pH 2.2 with HCl twice (5 min each) to effectively remove antibodies used for the first round of staining. Embryos were then incubated in anti-FLU antibody conjugated to AP (1:2000; 11426338910, Roche) and expression of *myl7* in 28 and 55 hpf embryos was then revealed by Fast Red (Sigma-Aldrich) staining. Embryos were post-fixed in 4% PFA, rinsed in PBST, and transferred to 95% glycerol. Whole-mount imaging of WISH-stained specimens was performed using a Leica DFC420C camera mounted on a Leica MZ16F stereomicroscope.

### Genotyping

Heat-shocked embryos were genotyped by PCR after completion of WISH as previously described (Shin et al., 2007). Embryos carrying the *hsp70l*:*bmp2b* transgene were identified by amplifying a segment overlapping on the *hsp70l* promoter and the *bmp2b* coding sequence (forward primer: 5′-CATGTGGACTGCCTATGTTCATC-3′; reverse primer: 5′-GAGAGCGCGGACCACGGCGAC-3′). Embryos carrying the *hsp70l*:*ca-fgfr1* transgene were identified by amplifying a portion of the DsRed coding sequence that is part of the transgenic cassette (forward primer: 5′-CTCCAAGGCCTACGTGAAGCAC-3′; reverse primer: 5′-CACGGGCTTCTTGGCCTTG-3′). Overactivation of BMP signaling in embryos obtained from outcrosses of heterozygotic Tg(*hsp70l:bmp2b*) founder fish caused a characteristic tail phenotype (ventralized) in about 50% of heat-shocked embryos when HS treatment was performed at 10 hpf. Genotyping showed that the tail phenotype clearly segregated with the presence of the *hsp70l:bmp2b* allele in all embryo clutches analyzed. If indicated, this phenotypic trait was used in some experiments to distinguish WT and *hsp70l:bmp2b* allele carriers when genotyping was difficult to perform.

### Zebrafish embryo whole-mount immunofluorescence

Whole-mount immunofluorescence (WIF) was essentially performed as previously described (Opitz et al., 2011). Briefly, PFA-fixed embryos were washed several times with PBST. Embryos were mildly permeabilized by proteinase K treatment, washed with PBST and incubated for 4-6 h in blocking buffer (PBST containing 10 mg/ml bovine serum albumin, 4% horse serum, 1% DMSO and 0.8% Triton X-100) at room temperature with gentle agitation. Embryos were then incubated in blocking buffer containing primary antibodies overnight at 4°C. After removal of primary antibody solutions, embryos were extensively washed in PBST and incubated in blocking buffer containing secondary antibodies overnight at 4°C. After removal of secondary antibody solutions, embryos were washed in PBST and incubated for 3 days in PBST containing Hoechst 33342 nuclear dye (1:7500) to counterstain cell nuclei. After several PBST washes, embryos were shortly post-fixed in 4% PFA for 20 min at room temperature and stored in PBST at 4°C until whole-mount imaging or vibratome sectioning.

The following antibodies were used: rabbit anti-Pax2a (1:250; GTX128127, GeneTex); mouse anti-E-cadherin (1:200; 610181, BD Transduction Laboratories); chicken anti-GFP (1:1000; ab13970, Abcam; also recognizes d2EGFP and YFP); rabbit anti-monomeric destabilized Kusabira Orange 2 (dmKO2; 1:250; PM051, MBL); mouse anti-dmKO2 (1:200; M168-3, MBL); Cy3-conjugated donkey anti-rabbit IgG (1:250; 711-165-152, Jackson ImmunoResearch); Cy3-conjugated donkey anti-mouse IgG (1:250; 715-165-150, Jackson ImmunoResearch); Alexa Fluor 647-conjugated donkey anti-rabbit IgG (1:250; 711-605-152, Jackson ImmunoResearch); Alexa Fluor 647-conjugated donkey anti-mouse IgG (1:250; 715-605-150 Jackson ImmunoResearch); Alexa Fluor 488-conjugated goat anti-chicken IgG (1:250; A-11039, Thermo Fisher Scientific).

For whole-mount imaging, WIF-stained specimens were embedded in 1% low melting agarose (Lonza) on Fluoro-Dish glass-bottom dishes (World Precision Instruments). Images were acquired using a Leica DFC7000T camera mounted on a Leica M165FC stereomicroscope and LAS X software (Leica).

### Confocal image acquisition and cell counting

Confocal microscopic analyses of the thyroid region of zebrafish embryos were performed on vibratome sections of WIF-stained specimens. After WIF staining, zebrafish embryos were embedded in 7% low melting agarose and cut into serial 100 µm thick sections on a vibratome. Sections were mounted in Glycergel (Dako) on custom-made #1.5 72×26 mm glass slides. Confocal images of zebrafish sections were acquired using an LSM510 confocal microscope (Zeiss) and Zen 2010 D software (Zeiss). For quantitative analyses of cell numbers, confocal *z*-stacks covering the whole thyroid region were acquired and the number of cells expressing specific markers was determined manually by analyzing consecutive *z*-stacks. Images were processed using ImageJ/Fiji with a 1-pixel radius minimum filter to remove artefacts.

### Mouse embryo WIF

For WIF labeling, embryos were incubated overnight in PBS-buffered blocking solution containing 1% bovine serum albumin (Sigma-Aldrich), 0.7% Triton X-100 (Sigma-Aldrich), 4% horse serum (Thermo Fisher Scientific) and 0.02% sodium azide (Sigma-Aldrich). Incubation with primary antibodies in blocking solution was performed for 3 days at 4°C with constant rocking followed by several 1 h washes in PBS. Incubation with secondary antibodies in blocking solution was performed for 2 days at 4°C with constant rocking followed by several washes in PBS. Antibodies used for IF were: rabbit anti-Pax8 (1:800; PA0300, Biopat Srl), rabbit anti-Nkx2-1 (1:800; PA0100, Biopat Srl), rat anti-E-cadherin (1:300; ab11512, Abcam), Alexa Fluor 488-conjugated donkey anti-rabbit IgG (1:250; 711-545-152, Jackson ImmunoResearch) and Cy3-conjugated donkey anti-rat IgG (1:250; 712-165-153, Jackson ImmunoResearch). To counterstain cell nuclei, whole embryos were incubated in DAPI solution (1:5000) for 2 days at 4°C. After further washing in PBS, stained specimens were postfixed in 4% PFA for 20 min at room temperature. Stained specimens were embedded in 7% low melting point agarose (Lonza) for vibratome sectioning (100-120 µm sections mounted in Glycergel) and sections were imaged using a Leica TCS SP8 confocal microscope.

## Supplementary Material

10.1242/develop.201023_sup1 Supplementary information
