# Supplementary material for: Mesodermal FGF and BMP govern the sequential stages of zebrafish thyroid specification
Source: Development. 2023 May 16;150(10):dev201023. doi: 10.1242/dev.201023 (PMC10214844; doi:10.1242/dev.201023)
Supplement: Supplementary information [file develop-150-201023-s1.pdf]

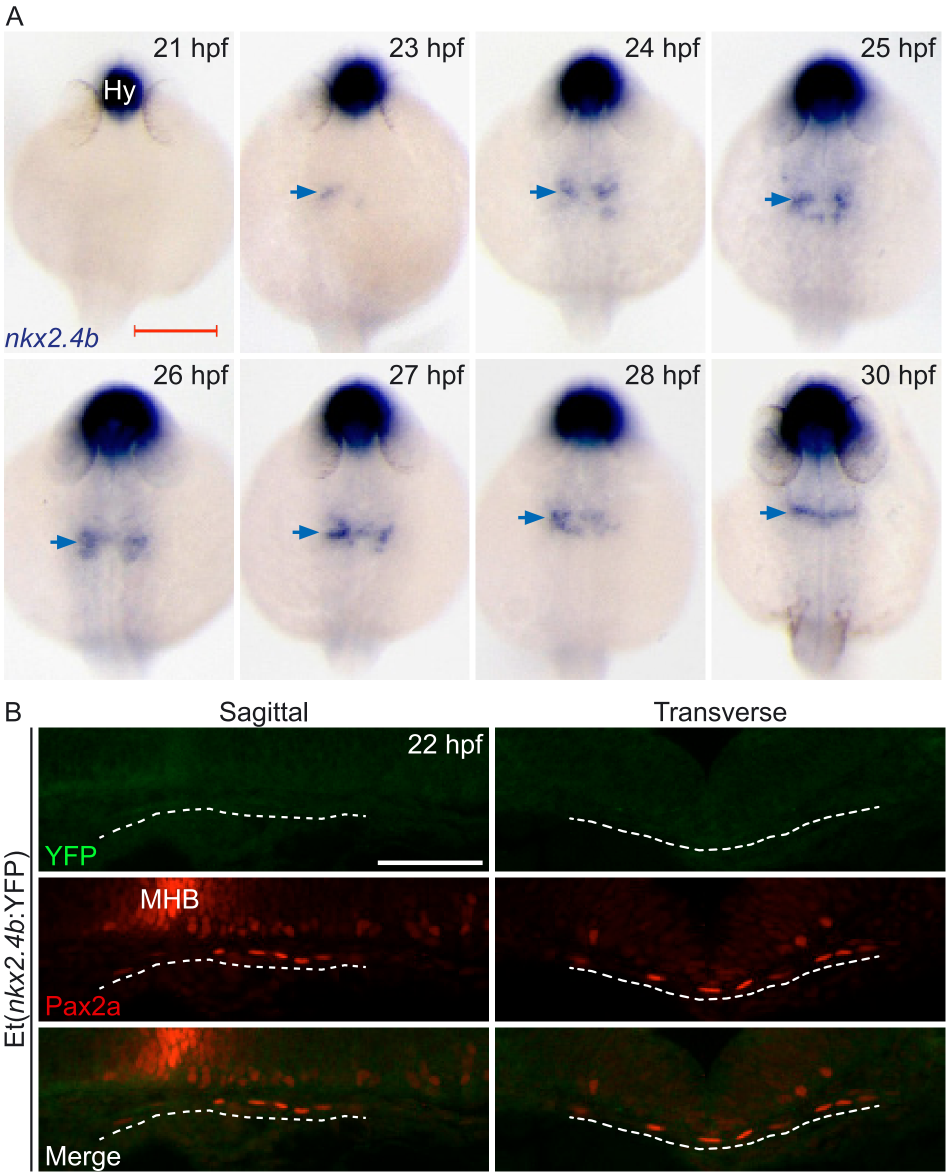

Fig. S1. Developmental expression of *nkx2.4b* mRNA in zebrafish embryos.

(**A**) Dorsal views of embryos after staining of *nkx2.4b* mRNA by whole-mount *in situ* hybridization, anterior is to the top. Note that *nkx2.4b* is expressed in the thyroid anlage (arrow) and the hypothalamus (Hy). Up to an embryonic age of 22 hpf (hours post-fertilization), *nkx2.4b* staining is restricted to the hypothalamus and is not detectable in the thyroid region (N=0/47 embryos). Expression of *nkx2.4b* mRNA in the prospective thyroid region can be first detected in 23 hpf embryos (N=32/56 embryos). At this stage, the *nkx2.4b* riboprobe weakly stained small clusters of cells located either unilateral (N=13/56 embryos) or bilateral (N=19/56 embryos) to the embryonic midline. 24 hpf is the earliest time point where robust *nkx2.4b* staining is detectable across all embryos examined (N=41/41 embryos). All embryos at 24 hpf and older stages show thyroidal *nkx2.4b* expression (N=187/187 embryos). From 24 to 26 hpf, thyroidal *nkx2.4b* staining was frequently detected in two seemingly separate domains located bilateral to the midline (N=58/81 embryos), with cells located close to the midline showing either weak or

no *nkx2.4b* staining. From 27 hpf throughout 30 hpf, the thyroidal *nkx2.4b* expression domain appeared more continuous (N=88/106), eventually presenting a mediolateral stripe-like morphology in most 30 hpf embryos (N=32/36 embryos). Scale bar: 200 μm. (**B**) Immunofluorescence of Pax2a and YFP in the thyroid region of Et(*nkx2.4b*:YFP) embryos at 22 hpf. Confocal images of transverse and sagittal (anterior to the left) sections show numerous Pax2a+ cells in the endoderm but YFP expression is undetectable at this stage. Dashed line depicts border between endodermal cell layer and foregut mesenchyme. Sagittal sections also highlight the anterior-posterior expansion of the Pax2a expression domain with the rostral edge positions ventral to the caudal portion of the midbrain-hindbrain boundary (MHB). The lack of YFP expression at 22 hpf in the majority of embryos analyzed at this stage correlates with the absence of Nkx2.4b mRNA staining in WISH analyses. Scale bar: 50 μm..

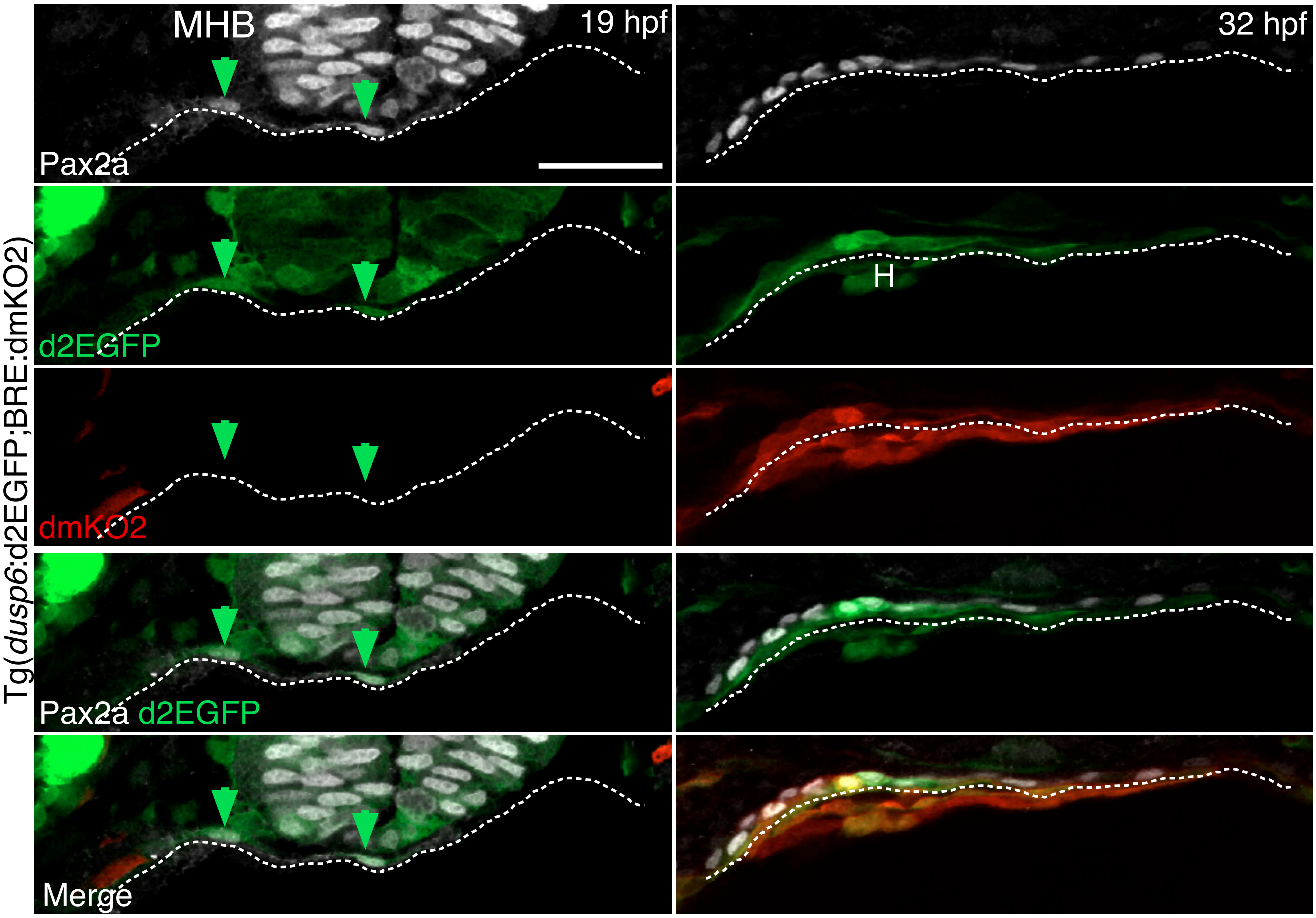

Fig. S2: Endogenous FGF and BMP signaling in Pax2a thyroid progenitors

Immunofluorescence of Pax2a, d2GFP and dmKO2 expression in anterior foregut of Tg(*dusp6*:d2EGFP;BRE:dmKO2) double transgenic embryos. Confocal images of transverse sections at the level of the forming thyroid anlage are shown. Dashed line depicts border between endodermal cell layer and ventral foregut mesenchyme. Note the absence of BMP signaling reporter expression (dmKO2) in endoderm and Pax2a+ cells at 19 hpf whereas endodermal FGF signaling reporter expression (d2GFP) is seen exclusively in the two Pax2a+ cells (arrowheads) that are present on this section. At 32 hpf, Pax2a+ cells show heterogenous d2GFP staining and a subset of Pax2a+ also shows dmKO2 staining. Note that endodermal BMP signaling reporter expression (dmKO2) is restricted to Pax2a+ cells. Pax2a+ cells located closest to the cardiac mesoderm (H, heart) display the strongest intensity of both FGF and BMP signaling reporter expression. MHB: midbrain-hindbrain boundary. Scale bar: 25 μm.

Development • Supplementary information

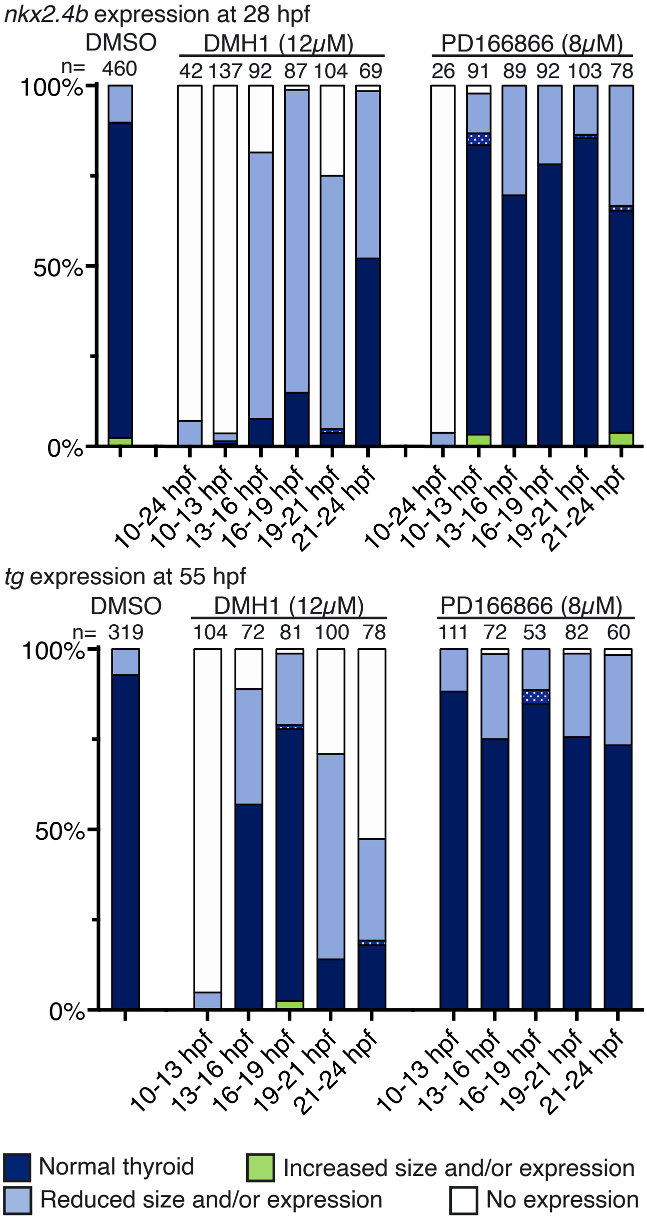

Fig. S3. Effects of timed inhibition of BMP and FGF signaling on thyroid marker expression

Distribution of thyroid phenotypes recovered by WISH at 28 hpf (*nkx2.4b* mRNA) and 55 hpf (*tg* mRNA) in embryos treated with inhibitors of BMP (12 μM DMH1) and FGF (8 μM PD166866) signaling during the indicated time intervals. Staining intensity of riboprobes for *nkx2.4b* and *tg* (*thyroglobulin*) was assessed manually and deviations from control embryos (0.1% DMSO) were classified into three main categories. Cases with abnormal morphology/positioning of thyroid marker domain are highlighted by dotted texture overlays.

Development • Supplementary information

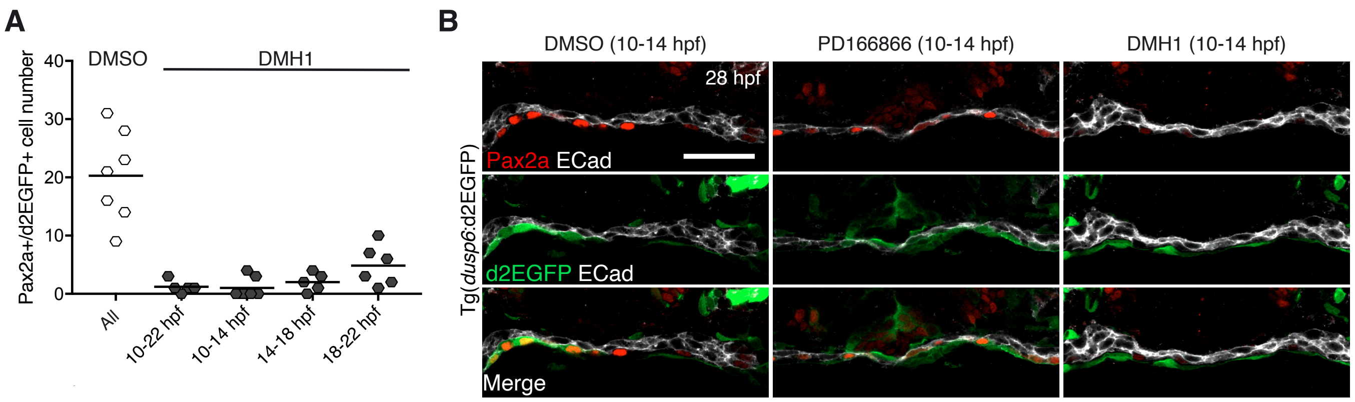

Fig. S4: Inhibition of BMP signaling affects endodermal FGF signaling at thyroid specification stages

(**A**) Quantification of the number of foregut Pax2a+ cells expressing the FGF signaling reporter d2EGFP in 28 hpf embryos treated with BMP signaling inhibitor for the indicated time intervals. Embryos of the Tg(*dusp6*:d2EGFP) FGF signaling biosensor line were treated with either 12 μM DMH1 or 0.1% DMSO (vehicle control) and analyzed by IF staining for Pax2a+ and d2EGFP expression at 28 hpf. Values of individual embryos are shown, bars depict mean values for each experimental group. (**B**) Immunofluorescence of Pax2a, E-cadherin (ECad) and d2EGFP expression in thyroid region of 28 hpf Tg(*dusp6*:d2EGFP) transgenic embryos treated with BMP and FGF signaling inhibitors during early somitogenesis (10-14 hpf). Confocal images of transverse sections are shown. Controls and PD166866-treated embryos (after 14 hours of drug washout) showed abundant endodermal Pax2a+ cells co-expressing the FGF signaling reporter d2EGFP. This population was absent in DMH1-treated embryos. Scale bar: 25 μm.

Development • Supplementary information

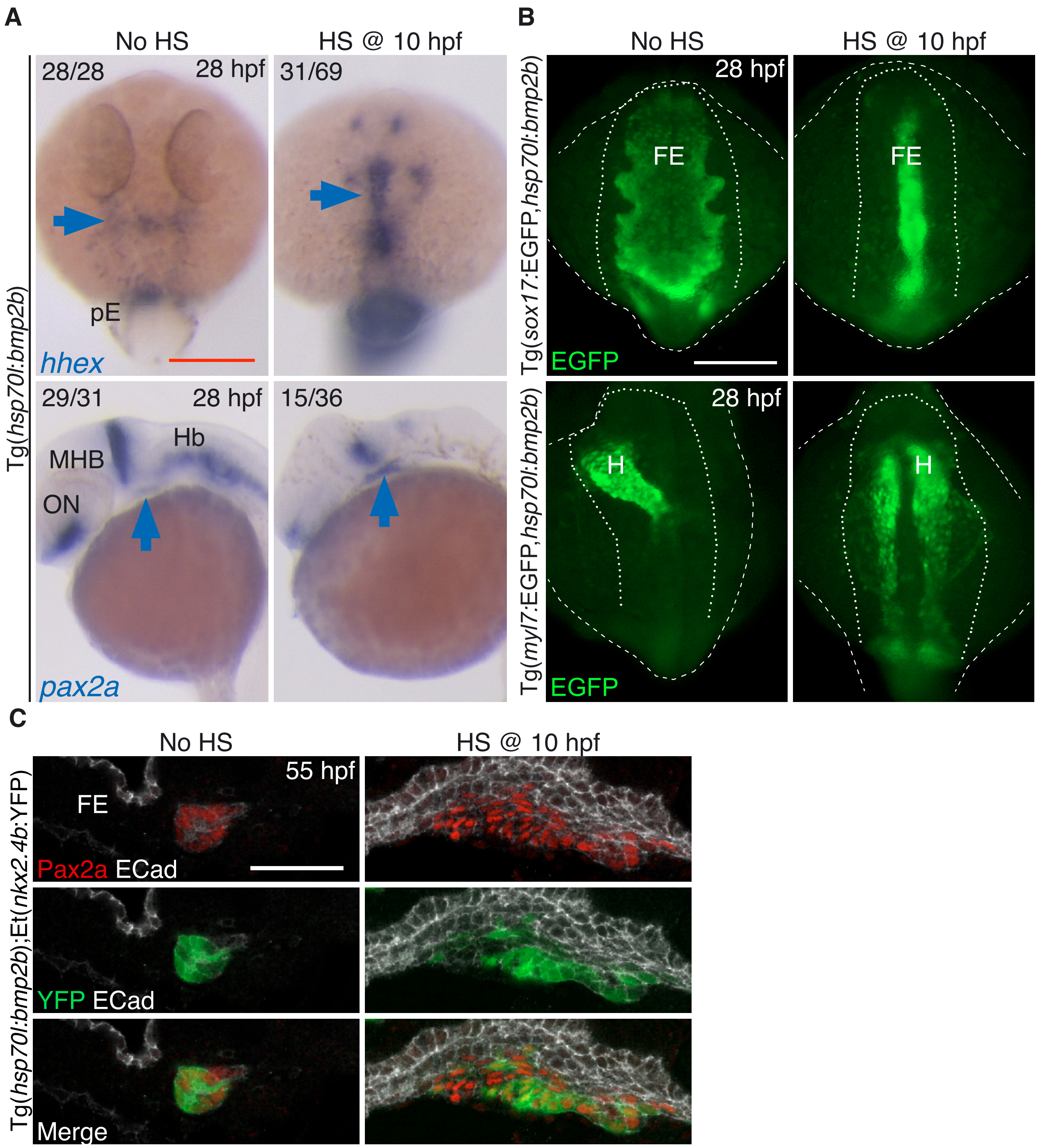

Fig. S5

Fig. S5. Global over-activation of BMP signaling during early somitogenesis promotes expansion of thyroid precursors

(**A**) Whole mount *in situ* hybridization of 28 hpf embryos for early thyroid markers *hhex* (dorsal views, anterior to the top) and *pax2a* (lateral views, anterior to the left) showed an aberrant enlargement of the thyroid anlage when heat shock (HS) treatment of Tg(*hsp70l:bmp2b*) embryos was applied at early somitogenesis. Note the caudally expanded expression thyroid domains of *hhex* and *pax2a*. Numbers indicate the proportion of embryos with the represented phenotype in the total number of observed embryos and include carriers and non-carriers of the

HS-inducible transgene. Hb: hindbrain; MHB: midbrain-hindbrain boundary; ON: optic nerve; pE: posterior endoderm. Scale bar: 200 μm. (**B**) Whole mount immunofluorescence of GFP reporter expression in Tg(*hsp70l*:*bmp2b,sox17*:EGFP) and Tg(*hsp70l*:*bmp2b,myl7*:EGFP) embryos (28 hpf) revealed severe maldevelopment of foregut endoderm (FE, upper panel) and cardiac mesoderm (H, heart; lower panel) if HS treatment was applied at 10 hpf. Note the abnormal rod-like appearance of the foregut endoderm and the failure of midline fusion and heart tube assembly of cardiomyocytes. Dorsal views are shown, anterior to the top. Scale bar: 200 μm. (**C**) Immunofluorescence of Pax2a, E-Cadherin (ECad) and YFP expression in thyroid primordium of 55 hpf double transgenic Tg(*hsp70l*:*bmp2b*),Et(*nkx2.4b*:YFP). Confocal images of sagittal sections are shown, anterior is to the left. Carriers of the HS-inducible *bmp2b* expression cassette showed a ventralized body morphology which facilitated rapid identification of transgene carriers. 55 hpf control embryos (no HS) showed formation of a small compact thyroid primordium that is already detached from the foregut epithelium. Stage-matched ventralized embryos showed the presence of a large mass of supernumerary Pax2a+/YFP+ cells that are not yet detached from a highly dysmorphic foregut epithelium. Scale bar: 25 μm.

Development • Supplementary information

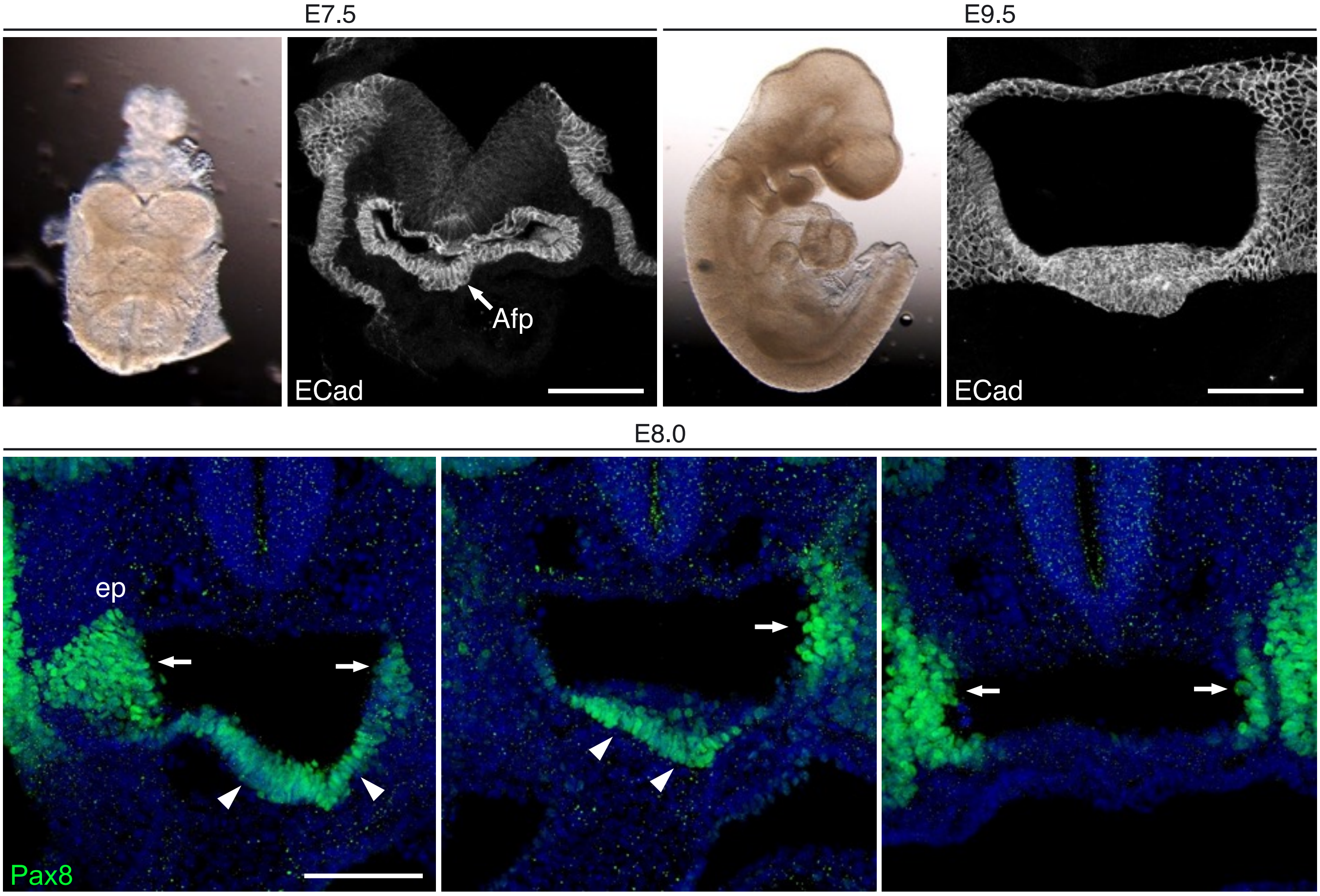

Fig. S6. Foregut morphogenesis and Pax8 expression in early mouse embryos

Upper panel shows the range of stages of mouse foregut development examined in this study. Embryos collected at embryonic day (E) E7.5 displaying a Theiler stage 12 morphology showed formation of a rudimentary anterior foregut portal (Afp). The 3D reconstruction of confocal images (E Cadherin IF staining) shows a 40 μm segment of the developing foregut. Embryos collected at E9.5 displaying a Theiler stage 15 morphology showed a well-developed foregut tube. The invagination of the ventral midline thyroid primordium has been initiated. The 3D reconstruction of confocal images shows a 90 μm segment of the anterior foregut at the level between the first and second branchial arch. The lower panel shows 3D reconstructions of confocal images (Pax8, DAPI) acquired from three consecutive 100 μm vibratome sections of a Theiler stage 13 embryo collected at E8.0. The rostral-most sections (left panel) show continuous Pax8 expression from ventral floor towards the endodermal pouch (ep) epithelium. The mid-sections show Pax8 expression in the ventral floor as well as in the lateral wall of the anterior foregut tube. The caudal-most sections (right panel) show Pax8 expression limited to lateral epithelium of the foregut tube. Arrowheads point to Pax8 expression in the ventral floor and arrows mark the lateral wall of the foregut. Scale bars: 100 μm.
