# Supplementary material for: Mesodermal FGF and BMP govern the sequential stages of zebrafish thyroid specification
Source: Development. 2023 May 16;150(10):dev201023. doi: 10.1242/dev.201023 (PMC10214844; doi:10.1242/dev.201023)
Supplement: Supplementary file 1 [file develop-150-201023_review_history.pdf]

Development

Mesodermal FGF and BMP govern the sequential stages of zebrafish thyroid specification

Benoit Haerlingen, Robert Opitz, Isabelle Vandernoot, Angelo Molinaro, Meghna Parakkal Shankar, Pierre Gillotay, Achim Trubiroha and Sabine Costagliola
DOI: 10.1242/dev.201023

Editor: Liz Robertson

Review timeline

Original submission:	14 June 2022
Editorial decision:	19 August 2022
First revision received:	1 March 2023
Editorial decision:	21 March 2023
Second revision received:	12 April 2023
Accepted:	13 April 2023

Original submission

First decision letter

MS ID#: DEVELOP/2022/201023

MS TITLE: Mesodermal FGF and BMP govern the multi-step thyroid specification

AUTHORS: Benoit Haerlingen, Robert Opitz, Isabelle Vandernoot, Angelo Molinaro, Meghna Shankar, Pierre Gillotay, Achim Trubiroha, and Sabine Costagliola

I have now received all the referees' reports on the above manuscript, and have reached a decision. The referees' comments are appended below, or you can access them online: please go to BenchPress and click on the 'Manuscripts with Decisions' queue in the Author Area.

As you will see, the referees express considerable interest in your work, but have some significant criticisms and recommend a substantial revision of your manuscript before we can consider publication. If you are able to revise the manuscript along the lines suggested, which may involve further experiments, I will be happy receive a revised version of the manuscript. Your revised paper will be re-reviewed by one or more of the original referees, and acceptance of your manuscript will depend on your addressing satisfactorily the reviewers' major concerns. Please also note that Development will normally permit only one round of major revision. If it would be helpful, you are welcome to contact us to discuss your revision in greater detail. Please send us a point-by-point response indicating your plans for addressing the referee's comments, and we will look over this and provide further guidance.

Please attend to all of the reviewers' comments and ensure that you clearly highlight all changes made in the revised manuscript. Please avoid using 'Tracked changes' in Word files as these are lost in PDF conversion. I should be grateful if you would also provide a point-by-point response detailing how you have dealt with the points raised by the reviewers in the 'Response to Reviewers' box. If you do not agree with any of their criticisms or suggestions please explain clearly why this is so.

Reviewer 1

Advance summary and potential significance to field

© 2023. Published by The Company of Biologists under the terms of the Creative Commons Attribution License (https://creativecommons.org/licenses/by/4.0/).

In this manuscript the authors investigate in vivo thyroid specification in zebrafish embryos. Key findings are the actual demonstration of a thyroid progenitor cell population of Pax2a positive cells characterized by enhanced FGF signaling and subsequent differentiation of cells from this progenitor cell pool to thyroid precursor cells, defined by the onset of Nkx2.4b expression under the influence of BMP signaling. This is a meticulously executed study that significantly contributes to our understanding of early events of thyroid specification. It might perhaps be argued that it does not uncover novel mechanisms pertaining to the specification of endodermally derived organs in general, given the well-established role of FGF and BMP signaling in early development of other endodermal derivatives. Furthermore, a role for FGF and BMP signaling in thyroid specification and development has for long been assumed. In recent years this notion has gained further support from studies on directed differentiation of stem cells to thyrocytes, as stimulation with FGF2 and BMP4 are integral components of such protocols as well as from a small-molecule screen in zebrafish embryos from the same authors as that of the present manuscript (Haerlingen et al 2019).
However, in my opinion this does not make the present manuscript less interesting and significant at all, as it is the first study that demonstrates that these signaling cues are instrumental to thyroid development also in vivo and furthermore adds an important temporal dimension. The finding of a temporal sequence of Pax2a and Nkx2.4b expression is significant as it provides the first actual correlate and molecular definition of the so far theoretical concept of thyroid progenitors and thyroid precursors.
On balance, in my opinion this manuscript indeed makes a significant and novel contribution to our understanding of developmental mechanisms.

Comments for the author

Major comments

- Detection of Pax2a and Nkx2.4b A major point of this manuscript is that Pax2a expression precedes that of Nkx2.4b and thus defines a thyroid progenitor cell pool. This conclusion rests on the finding that Pax2a expression can be detected by immunofluorescence with anti-Pax2a antibody staining earlier than that of Nkx2.4b as monitored by an enhancer trap line (Et(nkx2.4b:YFP)). As this is a key conclusion of this manuscript, I believe that it needs some further underpinning to be more convincing. Could it be that the detection threshold of Nkx2.4b (by the enhancer trap line) is higher than that for Pax2a detection (by antibody staining) and that this might explain why Pax2a expression seems to precede that of Nkx2.4b?
It would be good if the authors explain why they choose to detect Pax2a by direct antibody staining and Nkx2.4b in an indirect manned by using an enhancer trap line. The authors write that "… the YFP reporter expression neatly correlates with nkx2.4b mRNA expression during early zebrafish thyroid development … [data not shown]". I would prefer these data to be shown, perhaps as a supplemental figure. It is important to demonstrate that the trap line mirrors Nkx2.4 expression not only spatially, but also temporally (for instance by showing a comparison of Nkx2.4 ISH or IF with the enhancer trap line at different time points). It would be desirable if ISH data on Pax2a and Nkx2.4b are shown to further demonstrate that Pax2a expression precedes that of Nk2.4b.
Moreover, this key finding would be not only supported but of increased significance if it is shown that this temporal difference of expression is not exclusive to the zebrafish, but is also seen in other species. Indeed, in the discussion section the authors suggest that it is indeed the case also in mouse embryos and refer to it as "unpublished data". It would be clearly desirable if such mouse data were included in this manuscript. If it is shown that this temporal difference of expression is also seen in mouse thyroid development, it would greatly enhance the importance of this finding and make it even more convincing and exciting.

- Expression of putative ligands A key finding is that timed FGF and BMP signaling are instrumental to thyroid specification. The data presented in the manuscript indeed make a strong case for this notion and the authors suggest that the developing heart is a likely source of these signaling cues. It would by desirable if data on ligand (FGF and BMP) expression in the vicinity of the developing thyroid were included (at least for e.g. FGF2 and BMP4). This would provide more direct evidence for the model that is presented.
The close apposition of the developing thyroid to the mid/hindbrain boundary is striking in some of the figures. Only from viewing this spatial proximity it raises the question if signaling cues affecting the thyroid might be derived from this region. It would be interesting if the authors would comment on this possibility.

© 2023. Published by The Company of Biologists under the terms of the Creative Commons Attribution License (https://creativecommons.org/licenses/by/4.0/).

- Biosensor strains
The use of biosensor strains to map FGF and BMP activities is elegant. The fluorescent d2EGFP signal however seems somewhat weak. Could it be problematic to distinguish genuine positivity from background fluorescence? It would be desirable if some controls are shown as supplemental figures, for instance demonstrating that the biosensor signals (FGF and BMP) are increased after HS activating each pathway and/or decreased after treatment with small-molecule inhibitors.

- Statistics and interpretation
Key findings of the manuscript rely on manual cell counting and differences of cell numbers between different time point or conditions. In some instances it seems to me that the authors perhaps tend to overinterpret their data a bit and reach conclusions that are based on seemingly subtle differences of cell numbers. The only figure where statistical significance testing is presented is in Figure S2B. Why are p-values presented in this figure only, but not in the other graphs? Moreover, are the p-values calculated correctly in this figure?
This is of course difficult to tell from eyeballing only but is seems surprising that for instance in the DMH1 graph the mean cell number in the 10-14 hpf group is not significantly lower than in controls whereas it is indicated that the mean of the 18-22 hpf group is (at * significance level; albeit this group seems to contain fewer individuals) and also in the 22-26 hpf group (at *** significance level). Why is no significance testing presented for the other graphs of the paper?
In some instances, I am not sure that it is necessary, as only trends over time are discussed (e.g. Fig 1 and Fig 2). However, for figure/graph 4C the authors write that they "… observed an increase in the number of Pax2a+ endodermal cells in 22 and 28 hpf embryos …" and describe this as a "… robust shift towards higher Pax2a+ cell number…". I am aware of that genotyping was not possible and that a difference is likely obscured by the fact that the heat shocked embryos constitute a mix of embryos with and without the transgene, but at 22 hpf the differences between the groups seem very subtle. Is there a statistically significant increase of the mean cell number? It is also stated that "Short-term treatment with PD166866 had the strongest suppressive effect on Pax2a expression at late somitogenesis stages whereas transient inhibition of FGF signaling at early somitogenesis did not affect the number of Pax2a+ cells (Fig S2C). Should it be S2B? Moreover, the statement seems congruent with the medians, but at odds with the significance levels. Reasoning along the same lines goes also for Fig 5D (22 hpf).
For Fig 3 (nkx2.4b expression at 28 hpf) "BMP inhibitor" the authors write that "Inhibition of BMP signaling during mid-somitogenesis, i.e. from 13-16 hpf or 16-19 hpf caused only mild effects on thyroid marker expression. However applying DMH1 just prior to the concurrent onset of BMP signaling reporter and nkx2.4b expression, between 19 and 21 hpf, reduced or abolished nkx2.4b expression in almost all embryos". To me it is very difficult to appreciate any difference between the 13-16, 16-19 and 19-21 hpf bars, especially to argue that there is a difference between 13-16 hpf and 19-21 hpf. This might serve as an instance where, if I have understood the figure and text correctly, the data seem to perhaps be a bit overinterpreted. It either has to be shown in a statistically convincing way that such a difference is at hand or the interpretation and conclusion reached from it might need to be somewhat toned down.

- Readability
The section "Overactivation of BMP signaling induces supernumerary thyroid precursors" is heavy reading and occasionally a little bit difficult to follow.
It might be related to the fact that the effects of HS at 15 and 20 hpf are discussed first and HS at 10 hpf thereafter. I believe the authors have chosen to do so for good reasons (given the strong phenotype after HS at 10 hpf), but to the reader the complexity of this section is compounded by having to "go back" in the figures. It would be desirable if the authors could consider restructuring this section somewhat to make it more accessible to the reader.

- Selection of supplemental figures
In some instances, I do not really see the rationale for putting what seems to be figures illustrating key findings as supplemental figures, even though I understand that there might be space limitations. To me such key figures are Fig S1B and possibly Fig S5B, that are very illustrative and, in my opinion, would deserve to be included as regular figures.

© 2023. Published by The Company of Biologists under the terms of the Creative Commons Attribution License (https://creativecommons.org/licenses/by/4.0/).

Minor comments:

There are some typos that need to be corrected and instances of awkward phrasing that need to be revised, preferably by a native English speaker.

There is generally a lack of anatomical annotation in the microscopic images that might help the reader to understand structures, orientation, and topography. There are also some fluorescent structures that catch the eye of the reader but are not annotated or commented, making it difficult to understand if they are artifacts (e.g. Fig 1C; major green structure to the left in YFP 36 hpf, Fig 2C; intensely stained curved structure at bottom right in 22 hpf images).

Is annotation in Fig S1A lower left image correct? It is written “Pax2a dmKO2” but seems to be “Pax2a d2EGFP dmKO2” as all markers are visible in what seems to be a three channel merge (at 19 hpf but not at corresponding image at 32 hpf).

Lower panel of Fig S1B seems to lack annotation.

<u>Reviewer 2</u>

Advance summary and potential significance to field

In this manuscript by Haerlingen et al the authors study the mechanisms and signals that regulate the specification of thyroid precursor cells using the zebrafish embryo as a model. The authors observed a sequential activation of pax2b and nkx2.4b expression in endodermal cells that form the thyroid anlagen.
Using sensor lines to detect FGF and BMP signaling they find that before 20 hpf Pax2a expression is induced in endodermal cells with FGF signaling activity. BMP signaling activity was absent from these early Pax2b+ cells, but was observed at later stages in Pax2b+ cells which coincided with nkx2.4 expression. To study the role of FGF and BMP signaling they inhibited or ectopically activated FGF and BMP signaling at various time points and analysed pax2b and nkx2.4 induction in the endoderm and conclude a complex interaction between these signaling pathways during thyroid cell specification.

This study provides a detailed analysis of thyroid cell specification and identifies a complex role for FGF and BMP signaling in this process. Most novel aspect of the study is that the authors observe a sequential activation of pax2b and nkx2.4 expression in thyroid precursos cells which seems regulated by time depended activation of FGF and BMP signaling. The authors used of many transgenic lines to report and manipulate FGF and BMP signaling which is a clear strength of this study. The manuscript is well written and the images are of high quality. However, there are a number of issues and questions that the authors need to address before the manuscript can be accepted for publication which are listed below.

Comments for the author

Major concerns:

1. On page 7 the authors write: ‘Inhibition of BMP signaling during mid-somitogenesis, i.e. from 13-16 hpf or 16-19 hpf caused only mild effects on thyroid marker expression. However, applying DMH1 just prior to the concurrent onset of BMP signaling reporter and nkx2.4b expression, between 19 and 21 hpf, reduced or abolished nkx2.4b expression in almost all embryos.’

I don’t see this back in the data of figure 3 and S2. Effects of the 16-19h treatment seem comparable to effects of 13-16h treatment. How often have these experiments been repeated? Results from independent experiments will give insight into the variation of the results and will allow statistical analysis of the results.

2. On page 9 the authors write: ‘Importantly, overactivation of FGF signaling in double transgenic Tg(hsp70:cafgfr1);Et(nkx2.4b:YFP) embryos caused an increase in the number of YFP+ thyroid precursors at 28 hpf (Fig. 4C) but did not result in their premature differentiation(Fig. 4C)’

© 2023. Published by The Company of Biologists under the terms of the Creative Commons Attribution License (https://creativecommons.org/licenses/by/4.0/).

Based on which results do the authors conclude the latter (no premature differentiation).

3. In figure legends with heat shock experiments (Fig. 4-6) the authors write: 'Phenotypic data shown include carriers and non-carriers of the HS-inducible transgene.'

What does that mean? It suggests that the quantifications are based on mixtures of Tg carriers and non-carriers. So the question is: Are the phenotypic quantifications that are shown in these figures only based on embryos that carry the HS-transgene or is it based on a mixture of HS-transgenic and non-transgenic embryos? This should not happen as it gives a very distorted quantification as 50% of the embryos did not carry the HS-transgene. Based on what the authors write in the M&M where they state that embryos were genotyped after the WISH, I assume that this was done after scoring the embryos. However this is contradicting their statement in the figure legends. Please clarify

4. The results described and shown in Fig5 are not clear.

The authors write: Additional confocal analyses of IF-stained embryos (heat-shocked at either 15 or 20 hpf) revealed an increased number of Pax2a+/Nkx2.4b+ thyroid precursors at both 22 and 28 hpf in a morphologically intact foregut endoderm (Fig. 5D).

The graphs shown in 5D seem to display either pax2+ or YFP+ cells (not double labeled) as stated in the text. Moreover, the increase in either pax2+ or YFP+ cells is evident for the HS@10s but not for the other HS timepoints. How did the authors reach their conclusion?

The authors also write: Another key observation from these HS experiments was that enhanced BMP signaling failed to induce Nkx2.4b in all Pax2a+ cells so that Nkx2.4b expression was still restricted to only a fraction of endodermal Pax2a+ cells.

It is unclear were the data is displayed that would support this conclusion. I was expecting a graph similar to the one shown in Fig1B displaying the ratio of YFP/Pax2+ cells but could not find this.

5. Statistics: In the M&M the authors write that statistical analysis was done but the results of this cannot be found in the figures. The authors state that they have used a pairwise comparison which seems odd as multiple testing needs to be done for comparison of the quantification of the cell numbers with multiple timepoints. Please add the statistical analysis to all figures showing quantification of the data.

6. In the discussion the authors mention that the sequential expression of pax2 and nkx2.4 that they observed in zebrafish embryos was also observed in mouse embryos, however the data is not shown.
Inclusion of these data would strengthen the manuscript as it shows clear relevance to mammalian thyroid development.

Minor points:

Fig1. Please add a cartoon to show localisation of cross sections for non-zebrafish experts. Also a zoom-out to identify the MHB or other landmarks would be helpful.

Discussion: "apical pole of the heart tube' most likely refers to the arterial pole of the heart tube. Please correct.

First revision

Author response to reviewers' comments

Responses to reviewers

General comment on the use of statistical analyses in the revised manuscript
Both reviewers commenting on our manuscript raised certain concerns on our use of statistical

© 2023. Published by The Company of Biologists under the terms of the Creative Commons Attribution License (https://creativecommons.org/licenses/by/4.0/).

analyses for the complex data sets comprising in some transgene experiments data from a mix of transgene carriers and non-carriers without knowledge on the specific genotype of individual embryos. To clarify our methodology and data interpretation, we would like to note the following:
In all experiments with HS-inducible zebrafish lines, heterozygous HS carrier fish were crossed with either WT or Et(nkx2.4b:YFP) fish so that only 50% of the offspring would be carriers of the heat shock-inducible construct. This breeding scheme was necessary because homozygous HS carrier fish are very difficult to raise and maintain as adults.
When performing WISH analyses of such mixed populations of HS-transgene carriers and non-carriers, we always performed genotyping of a larger subset of WISH-stained embryos (but never for all, see high number of experimental embryos) after the phenotyping to correlate thyroid phenotypes with specific genotypes. In the case of WISH, genotyping post the phenotyping step was possible as sufficient tissue from stained specimen was available for DNA extraction and PCR-based genotyping.
We note that distribution graphs of phenotypes recovered based on WISH include not only the genotyped embryos but show data from all embryos analyzed (HS-transgene carriers and non-carriers). Representative images of WISH stained specimen are acquired from embryos with confirmed genotypes.
Our analysis of vibratome sections from WIF-stained embryos was more complicated. When analyzing WIF-stained embryos, we note that these embryos were completely sectioned and tissue sections were firmly mounted on slides and coverslipped. This technical step resulted in our inability to confirm the genotype (HS-carrier or not) of individual embryos. As a consequence, all our interpretations of cell number measurements (Pax2a+ as well as YFP+) are based on the assumption that the cohort of analyzed embryos contains up to 50 % transgene carriers. Because of this complex data structure, we decided to remove all statistical analyses from all data sets and present our data interpretation based on analyses of the distribution of cell number measurements. We also mention the data structure in the legends of all figures and in the corresponding sections of the main text.
When interpreting the cell number measurements in our revised manuscript, we focused attention on the distribution of individual cell number measurements and possible prominent shifts towards higher or lower values in a subset of analyzed specimen (tentative transgene carriers). Special attention was given to values in experimental groups that clearly fell out of the range of values determined control embryos. We also considered increased mean values as an additional indicator for HS-induced changes in cell number in a subset of embryos. Because of the uncertainty that is associated with this more tentative way of data interpretation, we re-phrased many of our conclusions to present a more balanced and cautious interpretation of our data.

Response to specific reviewer comments:

Reviewer #1
Major comments 1.
"A major point of this manuscript is that Pax2a expression precedes that of Nkx2.4b and thus defines a thyroid progenitor cell pool. This conclusion rests on the finding that Pax2a expression can be detected by immunofluorescence with anti-Pax2a antibody staining earlier than that of Nkx2.4b as monitored by an enhancer trap line (Et(nkx2.4b:YFP)). As this is a key conclusion of this manuscript, I believe that it needs some further underpinning to be more convincing. Could it be that the detection threshold of Nkx2.4b (by the enhancer trap line) is higher than that for Pax2a detection (by antibody staining) and that this might explain why Pax2a expression seems to precede that of Nkx2.4b? It would be good if the authors explain why they choose to detect Pax2a by direct antibody staining and Nkx2.4b in an indirect manned by using an enhancer trap line. The authors write that "… the YFP reporter expression neatly correlates with nkx2.4b mRNA expression during early zebrafish thyroid development … [data not shown]". I would prefer these data to be shown, perhaps as a supplemental figure. It is important to demonstrate that the trap line mirrors Nkx2.4 expression not only spatially, but also temporally (for instance by showing a comparison of Nkx2.4 ISH or IF with the enhancer trap line at different time points). It would be desirable if ISH data on Pax2a and Nkx2.4b are shown to further demonstrate that Pax2a expression precedes that of Nk2.4b. Moreover, this key finding would be not only supported but of increased significance if it is shown that this temporal difference of expression is not exclusive to the zebrafish, but is also seen in other species. Indeed, in the discussion section the authors suggest that it is indeed the case also in mouse embryos and refer to it as "unpublished data". It would be clearly desirable if such mouse data were included in this manuscript. If it is shown that

© 2023. Published by The Company of Biologists under the terms of the Creative Commons Attribution License (https://creativecommons.org/licenses/by/4.0/).

this temporal difference of expression is also seen in mouse thyroid development, it would greatly enhance the importance of this finding and make it even more convincing and exciting.."

Response: With respect to our experimental strategy to analyze the spatiotemporal patterns of Pax2a and Nkx2.4b by means of antibody staining of endogenous protein (Pax2a) versus antibody staining of a reporter protein (YFP) expressed in a transgenic line Et(nkx2.4b:YFP), we would like to state the following.

A central aim of our strategy was to utilize readouts that eventually would permit quantitative analyses of cell numbers for the different cell populations that we identified. A combination of anti-Pax2a and anti-Nkx2.4b antibodies would have been the ideal solution. Working in the zebrafish field, however, availability of specific antibodies is a very limited. The commercial antibody against zebrafish Pax2a protein was extensively validated in our lab including the use of pax2a KO fish (see Supplementary Figure 3 in our previous paper by Trubiroha et al. 2018). Over the years, we also dedicated/wasted quite substantial resources to buy and test a large number of proposed anti-Nkx2.4b antibodies; none of these antibodies ever worked in any specific manner. In the transgenic enhancer trap line Et(nkx2.4b:YFP), YFP expression is regulated by the zebrafish nkx2.4b promoter (information received from Becker lab in 2011; entry in ZFIN is ZDB-ALT-070410-22). We state this fact about regulation more prominently in the revised manuscript (line 135). We thus did not consider YFP detection as a problematic "indirect" detection method.

We would like to note that use of an antibody to detect YFP expression allowed for a first very dim staining in few 22 hpf cells while endogenous YFP fluorescence was undetectable before 24 hpf. Given the direct regulation of YFP expression by the *nkx2.4b* gene promoter, we reasoned that the antibody-based detection of YFP protein and the antibody-based detection of endogenous Pax2a protein does not introduce relevant confounding factors (such as proper YFP folding/maturation for emission of fluorescent light) when it comes to a comparative temporal expression analysis.

Now, a few words about our efforts on the alternative approach, meaning a comparative analysis of pax2a and nkx2.4b mRNA expression. At the developmental stages of concern (i.e. onset of pax2a expression between 19-22 hpf) the endodermal pax2a expression appears in close proximity to midbrain:hindbrain boundary (MHB) structures. This structure expresses pax2a mRNA at much higher levels and in many more cells.

ISH-based detection of pax2a in the MHB requires only 3 hours of incubation in staining solution to achieve a strong signal whereas staining of endodermal pax2a expression requires overnight incubation of embryos in staining solution. The very long incubation in staining solution required to label pax2a-expressing endodermal cells versus MHB cells results in a substantial "bleeding" of staining substrate from MHB structures into neighboring tissue including the ventrally located endoderm, thereby creating non-specific staining artefacts. It is for these reasons that we did not dare to base our conclusions about the differential onset of pax2a and nkx2.4b upon ISH staining.

With respect to temporal nkx2.4b mRNA expression, we can refer to a large body of literature (starting from the very initial description of nkx2.4b expression onset at 24 hpf in zebrafish thyroid by Rohr & Concha in 2000). Although different research groups used different riboprobes and staining protocols, there is no single report stating nkx2.4b mRNA expression before 22/23 hpf. For the purpose of the current study, we carefully stained several hundred of embryos (from many independent batches) with our nkx2.4b riboprobe and monitored stained specimen for the onset of first riboprobe staining. We present these WISH data in a new Fig. S1. Not a single embryo showed nkx2.4b mRNA staining prior to 22 hpf. The same conclusion holds true when we screened Et(nkx2.4b:YFP) embryos for YFP expression in the endoderm (see Fig. S1). The first few cells positively stained with our riboprobe appeared in some 22/23 hpf embryos. Thus, with respect to the temporal profile of nkx2.4b expression, data from WISH for nkx2.4b mRNA and antibody-based detection of YFP in Et(nkx2.4b:YFP) embryos are highly consistent.

Finally, to further foster the significance of our initial zebrafish findings, we now present a full data set on the temporal expression of Pax8 and Nkx2-1 during early foregut development (new Fig. 7 and new Fig. S6). We show that similar to zebrafish, Pax8 expression precedes the onset of Nkx2-1 expression. Strikingly, we observed first few Pax8+ cells already in the rudimentary anterior foregut portal of Theiler stage 12 embryos at E7.5. As description of embryonic age in embryonic days could be sometimes misleading when comparing data between studies, we preferred to present all our annotations of developmental age also by referring to Theiler stages. To firmly illustrate the developmental stage of embryos and foregut morphogenesis, we present additional data in a new Fig. S6. We also provide data showing not only an earlier onset of Pax8 expression but, similar to zebrafish, the existence of a supernumerary Pax8+ population in Theiler

© 2023. Published by The Company of Biologists under the terms of the Creative Commons Attribution License (https://creativecommons.org/licenses/by/4.0/).

stage 13 embryos collected at E8.0 (Fig. S6).
We agree with both reviewers that inclusion of the mouse data and the demonstration of a conservation from fish to mammals adds further relevance to our zebrafish work. We like to note, however, that we did not had the resources to recapitulate all the various zebrafish findings on the signaling pathways in a mouse model. We would be very glad if experienced mouse groups with access to appropriate genetic models could continue this work in the near future.

Major comments 2.
"A key finding is that timed FGF and BMP signaling are instrumental to thyroid specification. The data presented in the manuscript indeed make a strong case for this notion and the authors suggest that the developing heart is a likely source of these signaling cues. It would by desirable if data on ligand (FGF and BMP) expression in the vicinity of the developing thyroid were included (at least for e.g. FGF2 and BMP4). This would provide more direct evidence for the model that is presented. The close apposition of the developing thyroid to the mid/hindbrain boundary is striking in some of the figures. Only from viewing this spatial proximity it raises the question if signaling cues affecting the thyroid might be derived from this region. It would be interesting if the authors would comment on this possibility."

Response: The characterization of the spatiotemporal expression of the various FGF and BMP ligands that might be involved in conveying the mesoderm-to-endoderm signaling is a very interesting topic but it is also a very complex issue. We previously described the enhanced expression of zebrafish Bmp4 in cardiac mesoderm (Vandernoot et al., 2020, Fig. S9A) in very close proximity to the developing thyroid. With respect to FGF signaling, we previously reviewed the reported expression domains of FGF ligands based on the ZFIN (https://zfin.org/) and available phenotype data for LOF models thereof. As discussed in our previous report (Haerlingen et al. 2019), zebrafish *fgf8a* is a prime candidate based on its expression profile in precardiac and cardiac mesoderm (Reifers et al. 2000; PMID: 10603341) (https://zfin.org/ZDB- GENE-990415-72/expression). Previous studies have already shown that *fgf8a* mutants (ace) have impaired thyroid specification (Wendl et al., 2007). In addition to these two prime candidates, Bmp4 and Fgf8a, there are likely more ligands expressed but analyzing their expression across a range of relevant developmental stages is beyond our current capacities.

With respect to the notion that the MHB could also contribute signaling cues for thyroid precursor specification, we would like to add the following comments. At present, we cannot rule out this possibility but three arguments make this scenario less likely. First, it is only the most anterior portion of the Pax2+ domain that is positioned in very close proximity to the MHB, specifically the caudal portion of the MHB. When analyzing section from a rostral-to- caudal direction, the prominent MHB structures provided us with a very helpful morphological landmark to identify the rostral edge of the thyroid field. However, a large portion of the Pax2+ cells was positioned caudal to the MHB (this relative positioning is seen in sagittal sections of 22 hpf embryos in Figure S1B). Second, since the mouse Pax8 expression data indicate an evolutionary conservation of the sequential induction of thyroid TFs, it is of note that the Pax8 expression domain in mouse embryos is at no time in close vicinity to the neural tube. Third, the expanded thyroid field in ventralized embryos (generated by overactivation of BMP signaling at 10 hpf) is sandwiched between precardiac mesoderm but expands to positions at even greater distance relative to the MHB.

Major comments 3.
"The use of biosensor strains to map FGF and BMP activities is elegant. The fluorescent d2EGFP signal however seems somewhat weak. Could it be problematic to distinguish genuine positivity from background fluorescence? It would be desirable if some controls are shown as supplemental figures, for instance demonstrating that the biosensor signals (FGF and BMP) are increased after HS activating each pathway and/or decreased after treatment with small- molecule inhibitors"

Response: In zebrafish research, the Tg(dusp6:d2EGFP) line is the work horse when it comes to studies on FGF signaling. Beginning with the initial characterization by Molina et al. in 2007, this line has been intensively validated for its ability to report endogenous differences in FGF signaling activities but also to report the consequences of experimental blockade or over- activation of FGF signaling. Prominent recent examples include the life imaging studies by Roussigné et al (2018), PMID: 30282743 (see images on NCBI site).
With respect to the signal intensity, we would like to note that the d2EGFP reporter is a

© 2023. Published by The Company of Biologists under the terms of the Creative Commons Attribution License (https://creativecommons.org/licenses/by/4.0/).

destabilized version of EGFP with reduced half-live and the reporter intensity is therefore certainly lower than seen in reporters based on conventional GFP where accumulation of GFP over time results in high signal intensities. However, we intentionally selected the d2EGFP reporter to achieve a better temporal resolution. Moreover, the d2EGFP IF signal was in fact not so dim (as it might appear on some images) but several nearby cell populations including cells of the MHB and pharyngeal arches have a very strong d2EGFP expression (see for example high d2EGFP intensity in non-endodermal cell clusters in Figure S4B), that we had set the confocal imaging condition to a very low laser intensity. To capture regional and stage- dependent changes in FGF signaling, we applied the same imaging conditions to all tissue sections.

Major comment 4.
*"Key findings of the manuscript rely on manual cell counting and differences of cell numbers between different time point or conditions. In some instances it seems to me that the authors perhaps tend to overinterpret their data a bit and reach conclusions that are based on seemingly subtle differences of cell numbers. The only figure where statistical significance testing is presented is in Figure S2B. Why are p-values presented in this figure only, but not in the other graphs? Moreover, are the p-values calculated correctly in this figure? This is of course difficult to tell from eyeballing only but is seems surprising that for instance in the DMH1 graph the mean cell number in the 10-14 hpf group is not significantly lower than in controls whereas it is indicated that the mean of the 18-22 hpf group is (at * significance level; albeit this group seems to contain fewer individuals) and also in the 22-26 hpf group (at *** significance level). Why is no significance testing presented for the other graphs of the paper?"*

Response: We apologize for a major mistake that we made during the last steps of Figure processing. When rearranging graphs in this Figure (this figure is now Fig. 3), the annotation of statistical results for the two graphs of PD166866 and DMH1 effects were swapped, showing stats for DMH1 above the PD166866 graph and vice versa.
In the revised manuscript, we eventually decided to remove statistical analyses from all data sets for reasons outlined above.

"In some instances, I am not sure that it is necessary, as only trends over time are discussed (e.g. Fig 1 and Fig 2). However, for figure/graph 4C the authors write that they "… observed an increase in the number of Pax2a+ endodermal cells in 22 and 28 hpf embryos …" and describe this as a "… robust shift towards higher Pax2a+ cell number…". I am aware of that genotyping was not possible and that a difference is likely obscured by the fact that the heat shocked embryos constitute a mix of embryos with and without the transgene, but at 22 hpf the differences between the groups seem very subtle. Is there a statistically significant increase of the mean cell number?"

Response: We agree with the reviewer that some of our conclusions might be to strong given the subtle response and the uncertainty in genotype composition of the embryos analyzed. For the data set in question, we provide a more balanced data interpretation avoiding terms such as "robust shift". The wording reads now: *"IF analyses of heat-shocked embryos showed that HS treatment at 15 and 20 hpf increased the number of Pax2a+ endodermal cells in several embryos analyzed at 22 and 28 hpf (Fig. 4C). Although firm mounting of tissue sections on glass slides prevented PCR-based genotyping, we nevertheless regard the increased number of Pax2a cells in a subset of heat-shocked embryos as an indication that overactivation of FGF signaling promotes an increase in the number of Pax2a+ thyroid progenitors above the range observed in controls."*

It is also stated that "Short-term treatment with PD166866 had the strongest suppressive effect on Pax2a expression at late somitogenesis stages whereas transient inhibition of FGF signaling at early somitogenesis did not affect the number of Pax2a+ cells (Fig S2C). Should it be S2B? Moreover, the statement seems congruent with the medians, but at odds with the significance levels. Reasoning along the same lines goes also for Fig 5D (22 hpf)."

Response: The former Fig. S2B has been corrected and is now presented as regular Fig. 3. Thank you for bringing the mistakes to our attention.
"For Fig 3 (nkx2.4b expression at 28 hpf) "BMP inhibitor" the authors write that "Inhibition of BMP signaling during mid-somitogenesis, i.e. from 13-16 hpf or 16-19 hpf caused only mild effects on thyroid marker expression. However, applying DMH1 just prior to the concurrent onset of BMP signaling reporter and nkx2.4b expression, between 19 and 21 hpf, reduced or abolished nkx2.4b

© 2023. Published by The Company of Biologists under the terms of the Creative Commons Attribution License (https://creativecommons.org/licenses/by/4.0/).

expression in almost all embryos". To me it is very difficult to appreciate any difference between the 13-16, 16-19 and 19-21 hpf bars, especially to argue that there is a difference between 13-16 hpf and 19-21 hpf. This might serve as an instance where, if I have understood the figure and text correctly, the data seem to perhaps be a bit overinterpreted. It either has to be shown in a statistically convincing way that such a difference is at hand or the interpretation and conclusion reached from it might need to be somewhat toned down."

Response: We agree with the reviewer that the wording used in the original manuscript is difficult to align with the data shown. In the revised manuscript, we adopted a more balanced interpretation of our findings. For the specific section mentioned here, we now adopted the following wording: "Short-term DMH1 treatment from 10-13 hpf was as effective as DMH1 treatment throughout somitogenesis in completely abolishing nkx2.4b expression at 28 hpf whereas DMH1 treatments at later stages were generally less effective (Fig. 3A and Fig. S3). However, one remarkable finding was that a 2 hour treatment (from 19 to 21 hpf) with DMH1 just before the onset of endogenous BMP signaling and nkx2.4b mRNA expression greatly reduced or abolished nkx2.4b expression in 28 hpf embryos and also had a lasting impact on tg mRNA expression in 55 hpf embryos (Fig. 3A)."
From the various observations made in these phenotyping studies, we still would like to highlight that the fairly short 2 hour treatment resulted in not only reduced nkx2.4b expression but had a marked effect on the final formation of a thyroid primordium expressing the functional differentiation marker tg (thyroglobulin).
Another aspect as to why we pay special attention to the effects caused by treatment from 19 – 21 hpf is that most embryos often showed a very tiny cluster of weakly stained cells. Such embryos therefore fell into the category "reduced size and/or expression" although the reduced nkx2.4b staining indicated a more marked impairment of nkx2.4b mRNA expression compared to short treatments from 13-16 or 16-19 hpf (that were nonetheless counted in the same category).

Major comment 5.
"The section "Overactivation of BMP signaling induces supernumerary thyroid precursors" is heavy reading and occasionally a little bit difficult to follow. It might be related to the fact that the effects of HS at 15 and 20 hpf are discussed first and HS at 10 hpf thereafter. I believe the authors have chosen to do so for good reasons (given the strong phenotype after HS at 10 hpf), but to the reader the complexity of this section is compounded by having to "go back" in the figures. It would be desirable if the authors could consider restructuring this section somewhat to make it more accessible to the reader."

Response: We agree with the reviewer that this portion of our data presentation could be improved. For this reason, we split the presentation of results from HS experiments at 10 hpf and those from HS experiments later in somitogenesis. Separate presentation and interpretation of the very unique phenotypes observed during these different time points will hopefully improve the understanding of BMP effects. We first present data from the HS treatments at 10 hpf in a separate chapter "*Overactivation of BMP signaling in early somitogenesis induces supernumerary thyroid precursors*" and next present the data from the HS treatments at 15 & 20 hpf in another chapter "*Overactivation of BMP signaling in late somitogenesis accelerates thyroid precursor specification*". We hope that this re-organization improves the readability.

Major comment 6.
"In some instances, I do not really see the rationale for putting what seems to be figures illustrating key findings as supplemental figures, even though I understand that there might be space limitations. To me such key figures are Fig S1B and possibly Fig S5B, that are very illustrative and, in my opinion, would deserve to be included as regular figures."

Response: We authors also had several discussions what we seem critically important to be shown in the regular figures versus the supplemental figures. When revisiting the content, we actually agreed to move the former Fig. 3 to the supplementary material and instead show data from the former Fig. S3 in a new regular Fig. 3.

Minor comments (reviewer #1):
"There are some typos that need to be corrected and instances of awkward phrasing that need to be revised, preferably by a native English speaker."

© 2023. Published by The Company of Biologists under the terms of the Creative Commons Attribution License (https://creativecommons.org/licenses/by/4.0/).

Response: We rigorously revised wording and phrasing throughout the manuscript and hope that we could improve the quality of the revised manuscript in this regard.

There is generally a lack of anatomical annotation in the microscopic images that might help the reader to understand structures, orientation, and topography. There are also some fluorescent structures that catch the eye of the reader but are not annotated or commented, making it difficult to understand if they are artifacts (e.g. Fig 1C; major green structure to the left in YFP 36 hpf, Fig 2C; intensely stained curved structure at bottom right in 22 hpf images).

Response: We amended annotation in several figures to improve the orientation for readers not familiar with zebrafish development (a list of changes in figure artwork is appended at the end). Changes include for example the addition of a scheme highlighting the sectioning orientation in embryos plus a global overview of the anatomical organization in Fig. 1.
The major green structure to the left in YFP 36 hpf was annotated as hypothalamus (nkx2.4b expression domain)
The intensely stained curved structure at bottom right in 22 hpf images was labeled as part of cardiac structure in Fig 2C.

Is annotation in Fig S1A lower left image correct? It is written "Pax2a dmKO2" but seems to be "Pax2a d2EGFP dmKO2" as all markers are visible in what seems to be a three channel merge (at 19 hpf but not at corresponding image at 32 hpf).
Response: Thank you for bringing these mistakes to our attention. The Figure has been corrected.

Reviewer #2
Major comments 1.
"On page 7 the authors write: 'Inhibition of BMP signaling during mid-somitogenesis, i.e. from 13-16 hpf or 16-19 hpf caused only mild effects on thyroid marker expression. However, applying DMH1 just prior to the concurrent onset of BMP signaling reporter and nkx2.4b expression, between 19 and 21 hpf, reduced or abolished nkx2.4b expression in almost all embryos.'
I don't see this back in the data of figure 3 and S2. Effects of the 16-19h treatment seem comparable to effects of 13-16h treatment. How often have these experiments been repeated? Results from independent experiments will give insight into the variation of the results and will allow statistical analysis of the results."

Response: In line with similar concerns raised by reviewer #1, we adopted a more balanced interpretation of our findings. For the specific section mentioned here, we now adopted the following wording:
"Short-term DMH1 treatment from 10-13 hpf was as effective as DMH1 treatment throughout somitogenesis in completely abolishing nkx2.4b expression at 28 hpf whereas DMH1 treatments at later stages were generally less effective (Fig. 3A and Fig. S3). However, one remarkable finding was that a 2 hour treatment (from 19 to 21 hpf) with DMH1 just before the onset of endogenous BMP signaling and nkx2.4b mRNA expression greatly reduced or abolished nkx2.4b expression in 28 hpf embryos and also had a lasting impact on tg mRNA expression in 55 hpf embryos (Fig. 3A)."
From the various observations made in these phenotyping studies, we still would like to highlight that the fairly short 2 hour treatment resulted in not only reduced nkx2.4b expression but had a marked effect on the final formation of a thyroid primordium expressing the functional differentiation marker tg (thyroglobulin).
Another aspect as to why we pay special attention to the effects caused by treatment from 19 – 21 hpf is that most embryos often showed a very tiny cluster of weakly stained cells. Such embryos therefore fell into the category "reduced size and/or expression" although the reduced nkx2.4b staining indicated a more marked impairment of nkx2.4b mRNA expression compared to short treatments from 13-16 or 16-19 hpf (that were nonetheless counted in the same category).

Major comments 2.
"On page 9 the authors write: 'Importantly, overactivation of FGF signaling in double transgenic Tg(hsp70:cafgfr1);Et(nkx2.4b:YFP) embryos caused an increase in the number of YFP+ thyroid precursors at 28 hpf (Fig. 4C) but did not result in their premature differentiation(Fig. 4C)' Based on which results do the authors conclude the latter (no premature differentiation)."

© 2023. Published by The Company of Biologists under the terms of the Creative Commons Attribution License (https://creativecommons.org/licenses/by/4.0/).

Response: We thank the reviewer for bringing this sloppy wording to our attention. What we actually intended to highlight here is the fact that FGF overactivation at 15 hpf, for example, did not induce an earlier onset of detectable YFP expression, meaning no YFP precocious expression prior to 22 hpf. We changed the wording accordingly, the section now reads: "When performing a similar analysis in double transgenic Tg(hsp70:ca-fgfr1);Et(nkx2.4b:YFP) embryos, we observed that HS treatment at 15 and 20 hpf increased the number of YFP+ endodermal cells in many embryos analyzed at 28 hpf (Fig. 4C). We also noted that HS-induced overactivation of FGF signaling did not evoke an precocious onset of YFP expression as heat- shocked embryos still lacked YFP expression at 20/21 hpf (data not shown) and numbers of endodermal YFP+ cells in 22 hpf embryos were still low, similar to stage-matched controls (Fig. 4C)."

Major comments 3.
"In figure legends with heat shock experiments (Fig. 4-6) the authors write: 'Phenotypic data shown include carriers and non-carriers of the HS-inducible transgene.'
What does that mean? It suggests that the quantifications are based on mixtures of Tg carriers and non-carriers. So the question is: Are the phenotypic quantifications that are shown in these figures only based on embryos that carry the HS-transgene or is it based on a mixture of HS- transgenic and non-transgenic embryos? This should not happen as it gives a very distorted quantification as 50% of the embryos did not carry the HS-transgene. Based on what the authors write in the M&M where they state that embryos were genotyped after the WISH, I assume that this was done after scoring the embryos. However this is contradicting their statement in the figure legends. Please clarify"

Response: Please see our general comments on the use of statistics and the handling of data containing both transgene carriers and non-carriers above.

Major comments 4.
"The results described and shown in Fig5 are not clear.
The authors write: Additional confocal analyses of IF-stained embryos (heat-shocked at either 15 or 20 hpf) revealed an increased number of Pax2a+/Nkx2.4b+ thyroid precursors at both 22 and 28 hpf in a morphologically intact foregut endoderm (Fig. 5D). The graphs shown in 5D seem to display either pax2+ or YFP+ cells (not double labeled) as stated in the text. Moreover, the increase in either pax2+ or YFP+ cells is evident for the HS@10s but not for the other HS timepoints. How did the authors reach their conclusion?"

Response: First, we corrected all graph annotations. The top panels in Fig. 5D show measurements of Pax2a+ cell number. The bottom panels in Fig. 5D show the number of double positive Pax2a+/YFP+ cells.
With respect to our conclusion on BMP overactivation effects on cell numbers, we would like to note the following. Analysis of 22 hpf embryos showed that a subset of embryos heat- shocked at 10, 15 and 20 hpf contained a higher number of Pax2a+ as well as Pax2a+/YFP+ double positive cells. For both cell number measurements, we consider cell numbers above the range observed in controls as a very strong indicator for a HS-transgene effect. For the 28 hpf time point, we agree that such shift in the distribution is less prominent for embryos heat- shocked at 15 and 20 hpf although mean values were moderately increased.
Accordingly, we stated our conclusions from this experiment in a more balanced and cautious manner. It now reads: "*…the detection of increased Pax2a cell numbers (above the range detected in controls) in about half of all heat-shocked embryos analyzed at 22 hpf strongly indicates that BMP overactivation at 15 or 20 hpf induced an surplus of Pax2a+ thyroid progenitors at this stage. The stimulatory effect of BMP signaling on Pax2a cell numbers appeared to level off at 28 hpf (the stage when total Pax2a cell numbers peak in normal development) as heat-shocked embryos showed, if any, only small increases in Pax2a cell numbers (Fig. 5D). In the absence of more definitive genotyping data, we cautiously argue that enhanced BMP signaling at later somitogenesis stages accelerates the initial induction of Pax2a in the foregut (at 22 hpf) without strongly affecting the final size of the thyroid progenitor pool (at 28 hpf).*
Overactivation of BMP signaling at 15 and 20 hpf strongly accelerated the differentiation of Pax2a+/Nkx2.4b+ thyroid precursors at 22 hpf compared to controls (Fig. 5D). By 28 hpf, the number of Pax2a+/Nkx2.4b+ thyroid precursors was moderately increased in a subset of heat-shocked embryos, mirroring the moderate increase in nkx2.4b mRNA staining seen in genotyped

© 2023. Published by The Company of Biologists under the terms of the Creative Commons Attribution License (https://creativecommons.org/licenses/by/4.0/).

transgene carriers at 28 hpf (Fig. 5B)."

"The authors also write: Another key observation from these HS experiments was that enhanced BMP signaling failed to induce Nkx2.4b in all Pax2a+ cells so that Nkx2.4b expression was still restricted to only a fraction of endodermal Pax2a+ cells.
It is unclear were the data is displayed that would support this conclusion. I was expecting a graph similar to the one shown in Fig1B displaying the ratio of YFP/Pax2+ cells but could not find this."

Response: We arrived at this conclusion based on two observations. In individual confocal sections of the foregut of HS-treated embryos, we still observed that a number of Pax2a+ cells did not show co-expression of YFP. Typical examples of this pattern are shown in Fig. 5C.
In addition, the total number of Pax2a+/YFP+ double cells (shown in Fig. 5D, bottom panels) was clearly still much lower than the total number of Pax2a+ cells (shown in Fig. 5D, top panels). We added the corresponding references to images shown in Fig. 5C to the text.

Major comments 5.
"Statistics: In the M&M the authors write that statistical analysis was done but the results of this cannot be found in the figures. The authors state that they have used a pairwise comparison which seems odd as multiple testing needs to be done for comparison of the quantification of the cell numbers with multiple timepoints. Please add the statistical analysis to all figures showing quantification of the data."

Response: After several rounds of discussion among the authors of this paper and in response to the qualified criticism of the patchwork statistical approach used in the original submission, we decided to present the various cell number measurements without a statistical analysis. See also our response to comment 3 & 4.

Major comments 6.
"In the discussion the authors mention that the sequential expression of pax2 and nkx2.4 that they observed in zebrafish embryos was also observed in mouse embryos, however the data is not shown. Inclusion of these data would strengthen the manuscript as it shows clear relevance to mammalian thyroid development."

Response: We now included a substantial set of IF staining data in the MS, including a new Fig. 7 and an additional Fig. S6. See also our response to a similar comment made by reviewer #1.

Minor points.
"Please add a cartoon to show localisation of cross sections for non-zebrafish experts. Also a zoom-out to identify the MHB or other landmarks would be helpful."

Response: We added a scheme and a global view image in Fig. 1. to aid the interpretation of our data.
""apical pole of the heart tube' most likely refers to the arterial pole of the heart tube. Please correct."

Response: Thank you for bringing this error to our attention. Wording was changed accordingly.

Changes to the figures

- Figure 1:
    - Added schematic view of the embryo + large view of the imaged area
    - Added anatomy annotations (A and C)
    - Changed scale bar(s) colour from red to white
- Figure 2:
    - Added panel D (moved from Fig. S1B)
    - Added anatomy annotations (C and D)
    - Changed layout

© 2023. Published by The Company of Biologists under the terms of the Creative Commons Attribution License (https://creativecommons.org/licenses/by/4.0/).

  - Changed scale bar(s) colour from red to white
- Figure 3:
  - Swapped entirely with Fig. S2
  - Added color coded arrows (A)
  - Changed/added anatomy annotation (A and C)
  - Removed statistical analysis (B)
  - Changed scale bar(s) colour from red to white (C)
- Figure 4:
  - Changed anatomy annotations (B)
  - Updated legend to match data (A)
- Figure 5:
  - Updated legend to match data (A)
  - Changed anatomy annotation (B)
  - Change scales (D, lower panels)
  - Added ratio curve (E)
  - Updated scale (E)
  - Added n values above the first graph
  - Changed scale bar(s) colour from red to white (C)
- Figure 6:
  - Removed panel former panel A
  - Removed panel C (now Fig. 8)
  - Moved Fig. S5B into Fig. 6 as panel B. Removed panel A of Fig. S5.
  - Added anatomy annotations (all panels)
  - Added arrow (B)
- Figure 7:
  - Created Fig. 7 with mouse data
- Figure 8:
  - Move panel C from Fig. 6 as a new figure.
- Figure S1:
  - Created Fig. S1 as suggested by reviewers
  - All following supp. Figure number have been incremented by 1
- Figure S2:
  - Removed 31 hpf images
  - Moved panel B into Fig. 2
  - Added anatomy annotations
  - Corrected marker annotation (Pax2a dmKO2 □ Merge)
  - Exchanged lower right image for the correct one (showing all 3 markers instead of only Pax2a and dmKO2)
  - Changed scale bar(s) colour from red to white
- Figure S3:
  - Swapped entire figure with Fig. 3
- Figure S4:
  - Removed statistical analysis
  - Changed scale bar(s) colour from red to white
- Figure S5:
  - Added anatomy annotations
  - Changed scale bar(s) colour from red to white (B and C)
  - Adjusted brightness and contrast level and added dotter lines to better see embryos contours to increase clarity to non-zebrafish readers.
- Fig S6:
  - Created fig. S6

© 2023. Published by The Company of Biologists under the terms of the Creative Commons Attribution License (https://creativecommons.org/licenses/by/4.0/).

Second decision letter

MS ID#: DEVELOP/2022/201023

MS TITLE: Mesodermal FGF and BMP govern the sequential stages of zebrafish thyroid specification

AUTHORS: Benoit Haerlingen, Robert Opitz, Isabelle Vandernoot, Angelo Molinaro, Meghna Shankar, Pierre Gillotay, Achim Trubiroha, and Sabine Costagliola

I have now received all the referees reports on the above manuscript, and have reached a decision. The referees' comments are appended below, or you can access them online: please go to BenchPress and click on the 'Manuscripts with Decisions' queue in the Author Area.

The overall evaluation is very positive and we would like to publish a revised manuscript in Development. However as you will see Reviewer 2 has identified two very small points that they would like you to clarify in the manuscript - this should only take you a few minutes of work to alter the text, before uploading the final files. Your paper will not require any further re-review rather I will accept it once the final version has been received.

Please attend to all of the reviewers' comments in your revised manuscript and detail them in your point-by-point response. If you do not agree with any of their criticisms or suggestions explain clearly why this is so. If it would be helpful, you are welcome to contact us to discuss your revision in greater detail. Please send us a point-by-point response indicating your plans for addressing the referee's comments, and we will look over this and provide further guidance.

Reviewer 1

Advance summary and potential significance to field

In this manuscript the authors investigate in vivo thyroid specification mainly in zebrafish embryos, but in the revised verion to some extent also in mouse embryos. Key findings are the actual demonstration of a thyroid progenitor cell population of Pax2a positive cells characterized by enhanced FGF signaling and subsequent differentiation of cells from this progenitor cell pool to thyroid precursor cells, defined by the onset of Nkx2.4b expression under the influence of BMP signaling. This is a meticulously executed study that significantly contributes to our understanding of early events of thyroid specification. A role for FGF and BMP signaling in thyroid specification and development has for long been assumed. In recent years this notion has gained further support from studies on directed differentiation of stem cells to thyrocytes, as stimulation with FGF2 and BMP4 are integral components of such protocols as well as from a small-molecule screen in zebrafish embryos from the same authors as that of the present manuscript (Haerlingen et al 2019). In my opinion this does not make the present manuscript any less novel, interesting and significant at all, as it is the first study that demonstrates that these signaling cues are instrumental to thyroid development also in vivo and furthermore adds an important temporal dimension. The finding of a temporal sequence of Pax2a and Nkx2.4b expression is significant as it provides the first actual correlate and molecular definition of the so far theoretical concept of thyroid progenitors and thyroid precursors.
On balance, in my opinion this manuscript indeed makes a significant and novel contribution to our understanding of developmental mechanisms in thyroid and foregut development. It will most likely be a key reference not only in the field of thyroid developmental biology, but also for studies pertaining to the development of other endodermally derived organs.

Comments for the author

In think the authors have made a very thoughtful and careful revision of the manuscript. Their scientific rigour is evident both in the actual revision of the manuscript and figures as well as in their point-by-point rebuttal. In my opinion this has further improved an already fine mansucript, that obviously reflects a very large body of carefully executed and well designed experiments. As an example of this I want to highlight the inclusion of comparative data in the revised version on expression dynamics in mouse embryos, that clearly increases the general importance of this study.

© 2023. Published by The Company of Biologists under the terms of the Creative Commons Attribution License (https://creativecommons.org/licenses/by/4.0/).

I feel that all my points of criticism have been carefully and adequately addressed and that the manuscript is well suited for publication.

Reviewer 2

Advance summary and potential significance to field

This study provides a detailed analysis of thyroid cell specification and identifies a complex role for FGF and BMP signaling in this process. Most novel aspect of the study is that the authors observe a sequential activation of pax2b and nkx2.4 expression in thyroid precursos cells which seems regulated by time depended activation of FGF and BMP signaling. The authors used of many transgenic lines to report and manipulate FGF and BMP signaling which is a clear strength of this study. The manuscript is well written and the images are of high quality.

Comments for the author

The authors have addressed most of our questions in an adequate manner. There are a few minor points left to address:

1. In all figures showing images of WISH for which no quantification is shown (e.g Fig3A or Fig.6A,B), please indicate in the panels or in the legend how many embryos showed the reported expression.

2 Fig5A: are the embryos with a heat shock at 10-som sorted based on their ventralized phenotype? This would explain why the percentage of embryos with increased expression is >50%. However, in the legend it reads that the data includes carriers and non-carriers. Please explain this or correct when this is wrong.

Second revision

Author response to reviewers' comments

In the final version of our manuscript, we have incorporated the minor changes suggested by reviewer 2:

Comment: In all figures showing images of WISH for which no quantification is shown (e.g Fig3A or Fig.6A,B), please indicate in the panels or in the legend how many embryos showed the reported expression.
Answer: We have indicated in the legend of each figure the number of embryos with the reported phenotype and the total number of embryos analyzed.

Comment: Fig5A: are the embryos with a heat shock at 10-som sorted based on their ventralized phenotype? This would explain why the percentage of embryos with increased expression is >50%. However, in the legend it reads that the data includes carriers and non-carriers. Please explain this or correct when this is wrong.
Answer: added in the legend: "Numbers indicate the proportion of embryos with the phenotype shown out of the total number of embryos observed and include carriers and noncarriers of the HS - inducible transgene"

© 2023. Published by The Company of Biologists under the terms of the Creative Commons Attribution License (https://creativecommons.org/licenses/by/4.0/).

Third decision letter

MS ID#: DEVELOP/2022/201023

MS TITLE: Mesodermal FGF and BMP govern the sequential stages of zebrafish thyroid specification

AUTHORS: Benoit Haerlingen, Robert Opitz, Isabelle Vandernoot, Angelo Molinaro, Meghna Shankar, Pierre Gillotay, Achim Trubiroha, and Sabine Costagliola
ARTICLE TYPE: Research Article

I am happy to tell you that your manuscript has been accepted for publication in Development, pending our standard ethics checks.

© 2023. Published by The Company of Biologists under the terms of the Creative Commons Attribution License (https://creativecommons.org/licenses/by/4.0/).
